# Folliculin-interacting protein FNIP2 impacts on overweight and obesity through a polymorphism in a conserved 3′ untranslated region

**DOI:** 10.1186/s13059-022-02798-5

**Published:** 2022-10-31

**Authors:** Lara P. Fernández, Nerea Deleyto-Seldas, Gonzalo Colmenarejo, Alba Sanz, Sonia Wagner, Ana Belén Plata-Gómez, Mónica Gómez-Patiño, Susana Molina, Isabel Espinosa-Salinas, Elena Aguilar-Aguilar, Sagrario Ortega, Osvaldo Graña-Castro, Viviana Loria-Kohen, Pablo J. Fernández-Marcos, Alejo Efeyan, Ana Ramírez de Molina

**Affiliations:** 1 Molecular Oncology Group, IMDEA Food Institute, CEI UAM + CSIC, Madrid, Spain; 2 Spanish National Cancer Research Center, Madrid, Spain; 3 Biostatistics and Bioinformatics Unit, IMDEA-Food Institute, CEI UAM+CSIC, Madrid, Spain; 4 GENYAL Platform on Nutrition and Health, IMDEA Food Institute, CEI UAM + CSIC, Madrid, Spain; 5 Mouse Genome Editing Unit, Spanish National Cancer Research Center, Madrid, Spain; 6 Bioinformatics Unit, Spanish National Cancer Research Center, Madrid, Spain; 7 Institute of Applied Molecular Medicine (IMMA-Nemesio Díez), Department of Basic Medical Sciences, School of Medicine, San Pablo-CEU University, CEU Universities, Boadilla del Monte, Madrid, Spain; 8 Metabolic Syndrome Group, IMDEA Food Institute, CEI UAM + CSIC, Madrid, Spain

**Keywords:** Obesity, Overweight, Metabolism, mTOR, *FNIP2*, Folliculin complex, SNPs, miRNA

## Abstract

**Background:**

Overweight and obesity are defined by an anomalous or excessive fat accumulation that may compromise health. To find single-nucleotide polymorphisms (SNPs) influencing metabolic phenotypes associated with the obesity state, we analyze multiple anthropometric and clinical parameters in a cohort of 790 healthy volunteers and study potential associations with 48 manually curated SNPs, in metabolic genes functionally associated with the mechanistic target of rapamycin (mTOR) pathway.

**Results:**

We identify and validate rs2291007 within a conserved region in the 3′UTR of folliculin-interacting protein FNIP2 that correlates with multiple leanness parameters. The T-to-C variant represents the major allele in Europeans and disrupts an ancestral target sequence of the miRNA miR-181b-5p, thus resulting in increased *FNIP2* mRNA levels in cancer cell lines and in peripheral blood from carriers of the C allele. Because the miRNA binding site is conserved across vertebrates, we engineered the T-to-C substitution in the endogenous *Fnip2* allele in mice. Primary cells derived from *Fnip2* C/C mice show increased mRNA stability, and more importantly, *Fnip2* C/C mice replicate the decreased adiposity and increased leanness observed in human volunteers. Finally, expression levels of *FNIP2* in both human samples and mice negatively associate with leanness parameters, and moreover, are the most important contributor in a multifactorial model of body mass index prediction.

**Conclusions:**

We propose that rs2291007 influences human leanness through an evolutionarily conserved modulation of *FNIP2* mRNA levels.

**Graphical Abstract:**

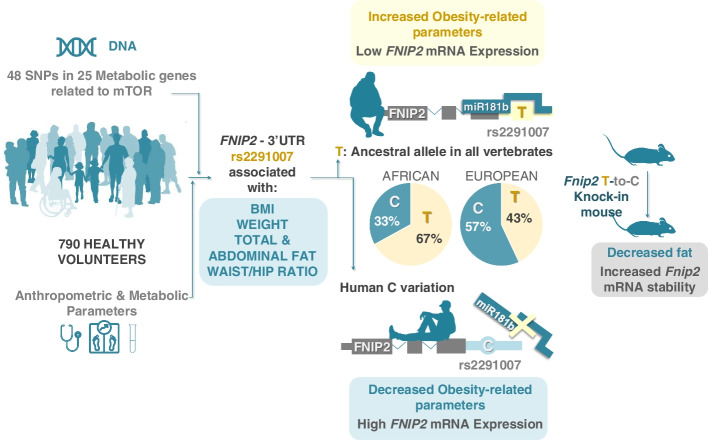

**Supplementary Information:**

The online version contains supplementary material available at 10.1186/s13059-022-02798-5.

## Background

Overweight and obesity are caused by an excessive and anomalous accumulation of adipose tissue and constitute a risk to health [1]. According to the World Health Organization (WHO), obesity is one of the most alarming health problems globally, excluding the sub-Saharan Africa and Asia. A body mass index (BMI, defined as the following ratio: weight (kg)/height (m)^2^) between 25 and 30 kg/m^2^ is considered overweight in adults, while a BMI exceeding 30kg/m^2^ is defined as obese state. Moreover, an elevated BMI strongly associates with several metabolic abnormalities, including insulin resistance and metabolic syndrome, themselves prominent risk factors for type 2 diabetes (T2D), cardiovascular diseases (CVDs) [2], and cancer [3, 4]. In addition, obesity and impaired metabolic health recently emerged as key risk factors for COVID-19 [5] and immune system dysfunction [6].

Both genetic and environmental factors contribute to the development of overweight and obesity. Lifestyle habits such as excessive caloric intake and insufficient physical activity are critical drivers of overweight and obesity [7]. In addition, genome-wide associations studies (GWAS), among other genetic approaches to define molecular drivers of obesity, have established strong associations between genetic variants and elevated BMI, being the fat mass and obesity-associated gene (*FTO*), the first genetic determinant of human body mass [8, 9]. A *G*enetic *I*nvestigation of *AN*thropometric *T*raits consortium (GIANT) meta-analysis (including more than 339,000 participants) linked a total of 97 loci with BMI, 56 of which were novel. The 97 loci account for 2.7% of BMI variation, and genome-wide studies estimate that common variants account for more than 20% of BMI variation [10–12]. Thus, genetic influence on BMI is still largely unknown, and in addition, most known associations have not been functionally validated.

It is not surprising that both genetic and environmental factors that contribute to obesity modulate cell signaling pathways responsive to nutrients and metabolic hormones. Among these pathways, the mechanistic target of rapamycin complex 1 (mTORC1) signaling pathway concurrently senses energy, nutrients, and growth factors and couples their sufficiency to the execution of anabolic cell growth and division [13]. MTORC1 itself is composed of the mTOR kinase and essential adaptor proteins (Raptor and mLST8), and dozens of proteins within several multimeric complexes participate in convergent regulatory cues that directly and indirectly control activation and inhibition of mTORC1. Substantial experimental evidence supports that mTORC1 controls several cellular and organismal processes that influence BMI, including food intake, insulin signaling, energy storage and consumption, metabolic activity, synthesis and secretion of hormones, and inter-organ communication [13–15]. Thus, it is reasonable to postulate that genetic variants affecting components of the mTORC1 pathway may contribute to determine overweight, obesity, and the associated abnormal metabolism.

While several studies have found single-nucleotide polymorphisms (SNPs) within components of the mTORC1 pathway affecting cancer susceptibility [16–18], mirror approaches establishing an association with increased BMI are awaited: to our knowledge, no genetic or epigenetic studies have linked SNPs in components of the mTOR pathway with overweight and obesity. The identification of such variants could pave the way toward tailored, precision therapeutic approaches targeting the mTORC1 pathway to combat the pathological states associated to elevated BMI.

Here, we measured BMI and multiple anthropometric and clinical features related to obesity in a cohort of 790 healthy volunteers and analyzed potential associations with 48 manually curated SNPs in metabolic genes with known functional associations to the mTORC1 pathway. We found an association between multiple metabolic parameters related to obesity and a human-specific SNP within an evolutionarily conserved region of the 3′UTR of the folliculin-interacting protein 2 (*FNIP2*) that affects mRNA stability. Such associations were replicated in a novel knock-in mouse strain genetically tailored to express the human-specific single-nucleotide variant. Moreover, in addition to the genetic association, the levels of *FNIP2* mRNA inversely correlate with anthropometric and clinical features of obesity. Thus, we propose that both rs2291007 and *FNIP2* blood mRNA levels are linked to obesity.

## Results

### *FNIP2* polymorphism rs2291007 is associated with metabolic and obesity-related phenotypes

We selected a total of 38 genes encoding proteins with metabolic functions linked to the mTOR pathway (Additional file 1: Fig. S1). We then manually curated potentially functional SNPs to perform a customized genotyping chip, according to the following criteria: SNPs located in coding regions, regulatory SNPs of 5′UTR or 3′UTR sites or SNPs in splicing sites. In addition, a threshold of 15% was established in European minor allele frequency (MAF).

We selected a total of 56 SNPs in 25 genes and genotyped them in a cohort of 790 healthy individuals. Their genomic positions are summarized in Additional file 2: Table S2. Evidence of departure from Hardy-Weinberg equilibrium (HWE) was observed for eight SNPs. They were excluded from the analysis, although none of them remained statistically significant after a conservative Bonferroni correction for multiple testing.

**Fig. 1:**
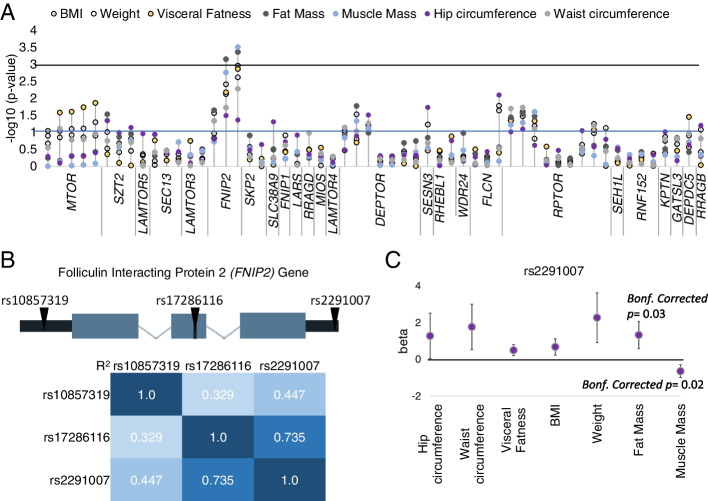
Genetic associations. **A** Representation of −log10 *p*-values for phenotypic associations with metabolic characteristics in 790 volunteers. Each point represents one SNP within a gene. Associations of the SNPs with body mass index (BMI), weight, visceral fatness, fat and muscle mass, and hip and waist circumference were modeled through linear regression on the SNP adjusted by sex and age. The −log10 *p*-values following an additive model for the SNPs (effect in the homozygote minor allele is twice as much as that of the heterozygote) were represented. **B** Gene location and linkage disequilibrium (LD) among rs10857319, rs17286116, and rs2291007. LD was calculated using LDLink (https://ldlink.nci.nih.gov/). **C** rs2291007 associations with several metabolic characteristics. Associations were modeled through linear regression on the rs2291007 adjusted by sex and age following an additive model. Ninety-five percent confidence intervals were used in beta estimates. Bonferroni (Bonf.) method was applied for multiple test correction of the *p*-values

Representation of −log10 *p*-values for additive model for each metabolic characteristic among the 790 Spanish volunteers are detailed in Fig. 1A and Additional file 1: Fig. S2. With a relatively permissive *p*-value threshold of 0.05, 33 SNPs in 17 genes were associated with 19 of 21 metabolic characteristics analyzed in our population, suggesting the relevance of genetic variations within selected genes in metabolic function and phenotype. Detailed information on rs number, genes, beta, 95% CI, and *p*-values for each metabolic association are summarized in Additional file 2: Table S3. Four SNPs located in three genes (two in *FNIP2* and one in each of *FLCN* and *RPTOR*) were associated with metabolic phenotypes (*FNIP2* with muscle and fat mass, visceral fatness, weight, BMI, and waist circumference; *FLCN* with hip circumference and *RPTOR* with systolic arterial pressure). Among these SNPs, the two located in *FNIP2* gene maintained statistical significance after applying the Bonferroni correction for multiple comparisons (Fig. 1A). Both were associated with fat mass (rs17286116, *beta*=1.31, 95% CI (0.55–2.07), Bonferroni-corrected *p-value*= 0.04 and rs2291007, *beta*=1.33, 95% CI (0.59–2.07), Bonferroni-corrected *p-value*= 0.03). rs2291007 was also associated with muscle mass (*beta*= −0.64, 95% CI (−0.99 to −0.29), Bonferroni-corrected *p-*value=0.02) (Fig. 1A).

We further analyzed the three SNPs detected in *FNIP2* genomic region (rs10857319, rs17286116, and rs2291007), located in 5′UTR, intronic, and 3′UTR regions, respectively (Fig. 1B and Additional file 2: Table S2). rs17286116 and rs2291007 share linkage disequilibrium, with a *R*^2^ value of 0.735 (Fig. 1B), explaining the overlapping associations detected. rs2291007, located in *FNIP2* 3′UTR region, had the highest *beta* and lowest *p*-values. Moreover, conditional analysis identified rs2291007 as the strongest independent signal for this locus, because adding the rs2291007 SNP to the analysis resulted in the other two SNPs (rs10857319 and rs17286116) losing statistical significance in predictive models for fat mass adjusted by sex and age. In addition to the associations with fat and muscle mass, the minor allele T of rs2291007 positively associated with several metabolic phenotypes, including weight and visceral fatness (*beta*= 2.27, 95% CI (0.91–3.62); *p*-value=0.001, Bonferroni-corrected *p-*value= 0.06 and *beta*= 0.5, 95% CI (0.2–0.81); *p*-value=0.001, Bonferroni-corrected *p-*value= 0.08 respectively). We also detected various association trends of rs2291007 with BMI, waist circumference, and hip circumference (*beta*= 0.68, 95% CI (0.24–1.12); *p*-value=0.002, *beta*= 1.77, 95% CI (0.53–3); *p*-value=0.005 and *beta*= 1.28, 95% CI (0.04–2.51); *p*-value=0.04 respectively) (Fig. 1C, Additional file 2: Table S3). No statistically significant correlations of rs2291007 with lipid or glucose profile and heart or nutritional parameters were found (Additional file 1: Fig. S2 and Additional file 2: Table S3).

In summary, our genetic study on 48 loci revealed that minor allele T of rs2291007 in *FNIP2* gene is linked to metabolic and obesity-related phenotypes, being associated with elevated fat mass, visceral fatness, weight, BMI, and waist and hip circumferences, and with decreased muscle mass in healthy individuals of European origin.

### miR-181b-5p selectively binds the 3′UTR of the *FNIP2* T allele

**Fig. 2:**
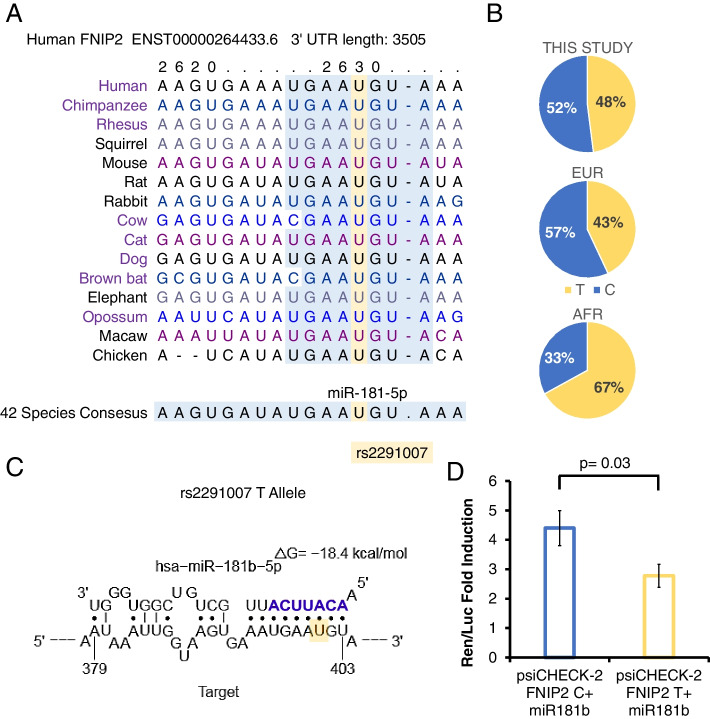
Binding of miR-181b-5p to the 3′UTR of the FNIP2 T allele. **A** TargetScanHuman predictions (http://www.targetscan.org/vert_72/) for rs2291007 genomic region. **B** rs2291007 frequencies across European (EUR) and African (AFR) populations (https://www.ensembl.org/). **C** Sfold (https://sfold.wadsworth.org/cgi-bin/index.pl) prediction for binding of miR-181b-5p to the rs2291007 region. **D** Dual-luciferase assays on psiCHECK vectors, for the binding of miR-181b-5p to rs2291007 region in presence of C or T alleles. Sequences for *FNIP2* 3′UTR with rs2291007 C or T alleles were cloned into psiCheck2 by ATUM. The relative luciferase activity (Renilla luminescence/firefly luminescence) was determined after transfection using the Dual-Luciferase Reporter assay system representing the translational repression of *FNIP2* upon binding to their 3′UTR of miR-181b-5p. Unpaired Student’s *t* test was used

SNP rs2291007 is a C/T variation in the 3′UTR of *FNIP2* and chromosome 4 open reading frame 45—*C4orf45*—gene, on chromosome 4. This genomic region is evolutionary conserved across vertebrates, as illustrated in Fig. 2A. The T allele in rs2291007 is ancestral and the common allele in the African population (Fig. 2B). In the European population, and in our study of healthy individuals with European origin, T is the minor allele of rs2291007 (Fig. 2B).

According to TargetScanHuman predictions (http://www.targetscan.org/vert_72/) [19], the sequence encompassing the SNP is a putative target region for miR-181b-5p binding (Fig. 2A). We in silico tested putative binding of miR-181b-5p to the rs2291007 region using Sfold (https://sfold.wadsworth.org/cgi-bin/index.pl) [20] (Fig. 2C). According to structural predictions, miR-181b-5p binds exclusively the T allele with two possible secondary conformations (Fig. 2C), and no sites for such binding are predicted for the C allele abundant in Europeans. Hence, only the T ancestral allele is potentially subjected to binding to, and thus, to regulation by, miR-181b-5p.

To validate the interaction of this miRNA with rs2291007 region, we performed dual-luciferase assays (Fig. 2D). In agreement with structural predictions, miR-181b-5p downregulated the activity of a reporter construction carrying the T, but not the C, allele. This result strongly suggests that miR-181b-5p selectively controls the expression of the T allele of *FNIP2* rs2291007 through binding to the 3′UTR.

### Levels of *FNIP2* mRNA associate with metabolic and obesity-related phenotypes

**Fig. 3:**
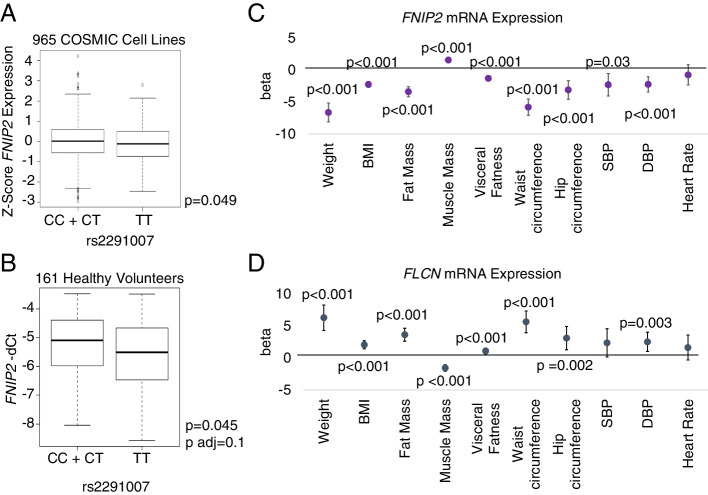
Transcriptomic consequences of rs2291007. **A** Box plot of the association between gene expression level for *FNIP2* and genotype for rs2291007. The box plot shows how the *FNIP2* expression values are distributed for each genotype from recessive model of inheritance in COSMIC cell lines. Common/major Homozygous plus Heterozygous alleles (C/C + C/T); Variant/minor Homozygous allele (T/T). Differences between gene expression in two groups were tested through Student’s *t* test. **B** Box plot of the association between gene expression level for *FNIP2* and genotype for rs2291007 in 161 healthy volunteers. The 2^−ΔΔCt^ method was applied to calculate the relative gene expression. Differences between gene expression in two groups were tested through Student’s *t* test and through linear models with adjustment variables. **C** Associations of *FNIP2* expression and phenotypic characteristics related to metabolism and obesity in 161 healthy volunteers. Differences between gene expression in two groups were tested through linear models with adjustment variables. All tests were bilateral. Ninety-five percent confidence intervals were used in beta estimates. Bonferroni method was applied for multiple-test correction of all *p*-values. **D** Associations of *FLCN* expression and phenotypic characteristics related to metabolism and obesity in 161 healthy volunteers. Differences between gene expression in two groups were tested through linear models with adjustment variables. All tests were bilateral. Ninety-five percent confidence intervals were used in beta estimates. Bonferroni method was applied for multiple-test correction of all *p*-values

Following on the observation that miR-181b-5p binds to the sequence containing the T allele of rs2291007, we sought evidence for a potential functional interaction between miR-181b-5p and rs2291007 to modulate the expression of *FNIP2*. First, we analyzed *FNIP2* mRNA levels in a panel of 965 Catalogue of Somatic Mutations in Cancer (COSMIC) cell lines [21] and found that rs2291007 minor homozygous cell lines (TT) (22.2%) displayed statistically significant decreased expression of *FNIP2*, as compared to cells carrying at least one copy of the C allele (77.8%) (Fig. 3A), consistently with the predicted loss of negative regulation by miR-181b-5p in the C allele.

Next, we aimed to confirm decreased *FNIP2* expression in carriers of the rs2291007 T allele by analyzing peripheral blood mononuclear cells (PBMC) of 161 healthy volunteers. Indeed, we found that homozygous TT individuals (25.46%) also showed lower *FNIP2* expression (*p-*value= 0.045) than individuals carrying at least one C allele (74.54%). When adjusted to sex and age, the associated *p*-value increased (*p-*value= 0.1) (Fig. 3B). We also analyzed the expression of *FNIP2* and *miR-181b-5p* in a subset of 89 healthy volunteers, and analysis of co-expression of *FNIP2* and *miR-181b-5p* showed an inverse correlation trend exclusively in carriers of T allele (*r*^2^=0.577, *p-*value=0.18 and *r*^2^=−0.777, *p*-value=0.35), while no trend was observed in carriers homozygous for the C allele (*r*^2^=0.004, *p*-value=0.99) (Additional file 1: Fig. S3A). These results support the existence of a functional relationship between miR-181b-5p and *FNIP2* expression by the selective binding to the T allele in rs2299007. We next analyzed the association between the expression of *FNIP2* and clinical parameters of healthy volunteers. Interestingly, and consistently with our expectations, the expression levels of *FNIP2* strongly associated with decreased weight, BMI, fat mass, visceral fatness, waist, and hip circumferences, systolic (SBP) and diastolic (DBP) blood pressure, blood levels of triglycerides and glycated hemoglobin, and low basal metabolism, and with increased muscle mass, all with high statistically significant *p*-values (Fig. 3C and Additional file 2: Table S4). These results support *FNIP2* expression in blood as a powerful marker of metabolic alterations.

Because FNIP2 is part of a heterotrimeric complex, we also analyzed expression of the other members, *FNIP1* and *FLCN*. *FNIP2* gene expression significantly correlated with both *FNIP1* and *FLCN* (Additional file 1: Fig. S3B) and we observed associations between the levels of *FLCN* expression and increased weight, BMI, fat mass, visceral fatness, waist and hip circumferences, DBP, and blood levels of glucose, leptin, and triglycerides; and decreased muscle mass (Fig. 3D and Additional file 2: Table S4). In contrast, we did not detect any relationship between the expression of *FNIP1* or *miR-181b* and phenotypic characteristics related to overweight or obesity (Additional file 2: Table S4).

### A knock-in mouse model for rs2291007

**Fig. 4:**
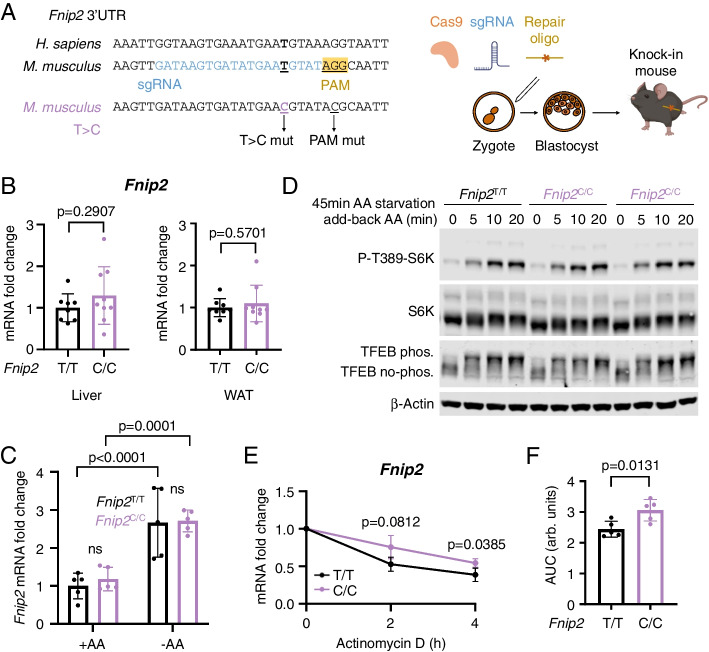
A *Fnip2*^C^ Knock-in mouse to model *FNIP2* rs2291007. **A** Experimental strategy to generate a knock-in mouse model mimicking *FNIP2* rs2291007C using CRISPR Cas9 gene-editing tool in blastocysts. **B** RT-qPCR from liver and gonadal white adipose tissue (WAT) obtained from *Fnip2*^T/T^ (*n*=8) and *Fnip2*^C/C^ (*n*=9) mice ad libitum fed. *Fnip2* mRNA levels are normalized to β-actin levels and relativized to the levels in *Fnip2*^T/T^. Statistical significance was calculated by using unpaired two-tailed *t*-test. **C ***Fnip2* mRNA levels were measured by RT-qPCR in mouse embryonic fibroblasts (MEFs) derived from *Fnip2*^T/T^ (*n*=5) and *Fnip2*^C/C^ (*n*=5) mice in complete Roswell Park Memorial Institute medium (RPMI; supplemented with dialyzed serum) or RPMI without amino acids for 2 h. *Fnip2* mRNA levels are normalized to β-actin levels and relativized to the levels in *Fnip2*^T/T^ +AA. **D** MEFs derived from *Fnip2*^T/T^ and *Fnip2*^C/C^ mice were deprived of all amino acids (AA) in RPMI supplemented with dialyzed serum for 45 min and re-stimulated with AA for 5, 10, and 20 min. Whole-cell protein lysates were immunoblotted for the indicated proteins. **E** MEFs derived from *Fnip2*^T/T^ (*n*=5) and *Fnip2*^C/C^ (*n*=5) mice were treated with 5μg/mL Actinomycin D for 2 and 4h. *Fnip2* mRNA levels were measured by RT-qPCR, normalized to β-actin levels, and then made relative to *Fnip2* levels in untreated samples. Statistical significance was calculated using 2-way ANOVA with Sidak’s multiple comparison correction. **F** The area under the curve (AUC) was calculated from **E** and statistical significance was calculated by using unpaired two-tailed *t*-test

The ancestral T allelic variant is evolutionary conserved in mammals (Fig. 2A) and present in the 3′UTR region of mouse *Fnip2* together with the surrounding seed region for miRNA-181b-5p (Fig. 4A). Thus, we decided to genetically engineer the C allelic variant in the mouse genome and to assess its functional impact on mouse weight and fat content. We used CRISPR/Cas9 genome engineering [22] in mouse zygotes to knock-in the T-to-C change. For technical reasons, we substituted one additional nucleotide in +6 position to disrupt the PAM sequence (a G-to-C change) so as to prevent recognition by CRISPR/Cas9 and unwanted sequence retargeting (Fig. 4A). Importantly, this additional change does not alter the seed region nor the predicted binding of miR-181b-5p (Additional file 1: Fig. S4A). Blastocytes of pure C57BL/6 background have raised to founder targeted chimeras, and *Fnip2*^T/T^, *Fnip2*^T/C^, and *Fnip2*^C/C^ mice were obtained at the expected Mendelian ratios from heterozygous breeders (Additional file 1: Fig. S4B). Macroscopically, *Fnip2*^C/C^ and *Fnip2*^T/C^ knock-in mice were indistinguishable from those expressing the ancestral T allele in homozygosity.

There are no commercially available antibodies against mouse Fnip2 to assess a potential difference in Fnip2 protein levels in *Fnip2*^T/T^ versus *Fnip2*^C/C^ cells and organs, so we first measured the levels of *Fnip2* mRNA in liver and gonadal WAT samples from ad libitum fed and 16-h-fasted mice. In contrast to the positive association between the C allele and steady-state levels of *FNIP2* mRNA observed in human cancer cell lines and in PBMC from healthy volunteers (Fig. 3), we found no association in mouse tissues (Fig. 4B and Additional file 1: Fig. S4C). We next obtained *Fnip2*^T/T^ and *Fnip2*^C/C^ mouse embryonic fibroblasts (MEFs) and quantified the levels of *Fnip2* mRNA in complete culture medium and in medium without amino acids to modulate the expression of *Fnip2*. Although we observed the expected increase in *Fnip2* mRNA levels upon amino acid deprivation, there was no difference between the levels of *Fnip2* mRNA in *Fnip2*^T/T^ and *Fnip2*^C/C^ MEFs (Fig. 4C). Consistently, the regulation of the mTORC1 pathway upon amino acid deprivation and stimulation, revealed by the phosphorylation of S6K1 in threonine 389, and by the upshift in the band corresponding to total levels of the transcription factor EB (TFEB) band caused by mTORC1-dependent phosphorylation, were indistinguishable between *Fnip2*^T/T^ and *Fnip2*^C/C^ MEFs (Fig. 4D). The absence of evidence for an increase in the mRNA levels in mouse cells expressing the C allele in homozygosity in the assayed conditions does not rule out that, under specific perturbations, such difference may exist; but to exclude the possibility that similar mRNA levels of *Fnip2* may be a consequence of lack of expression of the miR-181b-5p, we measured its levels in cells cultured with and without amino acids for 2 h. miR181b-5p was detected at similar levels in *Fnip2*^T/T^ and *Fnip2*^C/C^ MEFs in both culture conditions (Additional file 1: Fig. S4E). Alternatively, if miR181b-5p is present, a prediction is that the C variant should be more stable than the T variant, regardless of other compensatory mechanisms that may obscure such difference under steady-state synthesis of *Fnip2* mRNA. Thus, we halted new synthesis of all mRNA with Actinomycin D, inhibitor of RNA polymerase II activity, and measured the relative decay of *Fnip2* mRNA levels in *Fnip2*^T/T^ and *Fnip2*^C/C^ MEFs. Consistently with impaired binding of the *Fnip2*^C^ variant to miR-81b-5p, the decay of *Fnip2* mRNA levels was significantly slower in *Fnip2*^C/C^ cells, in comparison to that of *Fnip2* mRNA in *Fnip2*^T/T^ cells (Fig. 4E, F), indicating an increased stability of *Fnip2* mRNA in presence of rs2291007 C allele upon an abrupt interruption of new mRNA synthesis. In summary, the T-to-C substitution in mouse *Fnip2* does not have obvious effects on mTORC1 activity in the conditions assayed, neither does it lead to a detectable increase in *Fnip2* mRNA in steady state, but results in an increased stability of the *Fnip2* mRNA, a result that supports a disrupted binding of miR-181b-5p to the *Fnip2*^C^ variant.

**Fig. 5:**
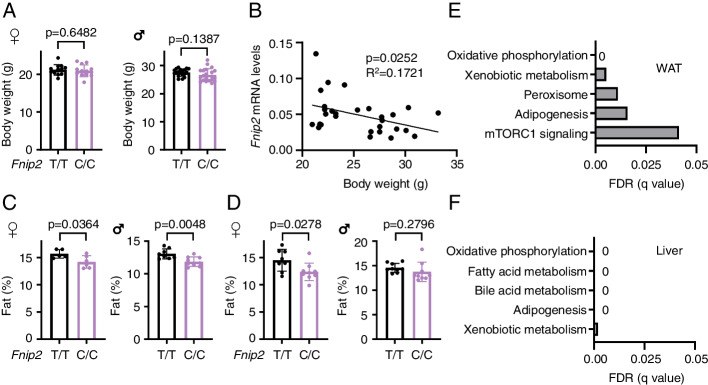
Impact of the engineered T-to-C substitution on mouse adiposity. **A** Body weight of 11–15-week-old *Fnip2*^T/T^ (*n*=12) and *Fnip2*^C/C^ (*n*=13) females and 10–13-week-old *Fnip2*^T/T^ (*n*=19) and *Fnip2*^C/C^ (*n*=21) males. **B** Correlation between *Fnip2* mRNA levels in livers from *Fnip2*^T/T^ (*n*=14) and *Fnip2*^C/C^ (*n*=15) mice and their body weight. **C** Fat content was obtained by measuring body composition in 5–6-week-old *Fnip2*^T/T^ (*n*=5) and *Fnip2*^C/C^ (*n*=6) females and *Fnip2*^T/T^ (*n*=8) and *Fnip2*^C/C^ (*n*=9) males. **D** Fat content was obtained by measuring body composition in 11–15-week-old *Fnip2*^T/T^ (*n*=8) and *Fnip2*^C/C^ (*n*=9) females and 10–13-week-old *Fnip2*^T/T^ (*n*=9) and *Fnip2*^C/C^ (*n*=9) males. In all cases, statistical significance was calculated using an unpaired two-tailed *t*-test

As mentioned, *Fnip2*^C/C^ mice were viable, macroscopically indistinguishable, and fertile. While no differences in body weight were seen between adult *Fnip2*^T/T^ and *Fnip2*^C/C^ mice (Fig. 5A), we observed a negative correlation between mRNA levels of *Fnip2* and mouse body weight (Fig. 5B), in sharp consistency with the association found in human samples (Fig. 3C). Such negative association with body weight was exclusive for *Fnip2*, as we saw no statistically significant association between the mRNA levels of the other two components of the Folliculin complex, *Fnip1* and *Flcn*, and body weight (Additional file 1: Fig. S5A). Nevertheless, a positive association on the mRNA levels of the three components of the complex (*Fnip2*, *Fnip1*, and *Flcn*) was observed (Additional file 1: Fig. S5B). Strikingly, in agreement with the association found in healthy volunteers (Fig. 1A, C), a significant decrease in the fat content was recapitulated in 5–6-week-old *Fnip2*^C/C^ male mice, and in female *Fnip2*^C/C^ mice analyzed at 5–6 and 11–15 weeks of age (Fig. 5C, D), and a similar trend was detected in 1-year-old females (Additional file 1: Fig. S5C). This difference does not seem to occur by a selective decrease on specific WAT depots, but from a general reduction in both visceral and subcutaneous depots (Additional file 1: Fig. S5D). To further investigate the consequences of the expression of the *Fnip2*^C^ variant in mice, we performed a transcriptomic analysis of two metabolically relevant organs, liver, and visceral WAT, from *Fnip2*^T/T^ and *Fnip2*^C/C^ mice. Strikingly, while only 9 genes were differentially expressed in livers, 4795 genes were differentially expressed in WAT from *Fnip2*^C/C^ versus *Fnip2*^T/T^ mice, indicating a comparably larger effect of the expression of the SNP in *Fnip2* in adipose tissue, as compared to liver. We next conducted Gene Set Enrichment Analyses (GSEA) in samples from *Fnip2*^C/C^ versus *Fnip2*^T/T^ livers and WAT. Strikingly, among the top 5 signatures identified in both liver and WAT analyses, “adipogenesis,” “fatty acid metabolism,” and “mTORC1 signaling” were enriched samples from of *Fnip2*^T/T^ mice (Fig. 5E, F; Additional file 1: Fig. S5E and S5F), consistently with the increased fat content observed in *Fnip2*^T/T^ mice.

Thus, altogether, the mouse genetic data strongly support that the 3′UTR of *FNIP2* is an evolutionary conserved genetic determinant of lean-fat mass ratio.

### Multifactorial genetic model for overweight and obesity risk

**Fig. 6:**
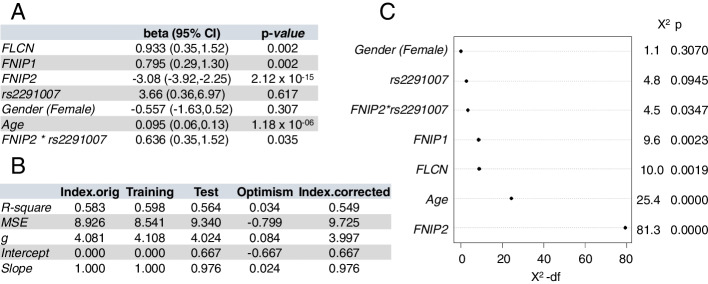
Multifactorial genetic model for overweight and obesity risk. **A** Multifactorial model to predict body mass index (BMI). Estimated parameters and 95% CI, plus *p*-values of variables in the multifactorial model. The linear regression model included the rs2291007 SNP (in additive form), three gene expression variables (*FNIP2*, *FNIP1*, and *FLCN*), plus sex, age, and the interaction between *FNIP2* gene expression and rs2291007. **B** Bootstrap validation of the multifactorial model. Multifactorial model was validated through bootstrap validation, using 2000 bootstrap samples, which correction by optimism (MSE, Mean Square Error). **C** Variable importance plot for the multifactorial model, using *χ*^2^ – df (degrees of freedom) as metrics for the importance of each variable

Overweight and obesity are complex conditions modulated by several causes, so we designed a multifactorial model to predict BMI taking into consideration the relevance of genetic susceptibility of the FLCN-FNIP complex investigated herein. We derived a linear regression model that included the rs2291007 SNP (in additive form), three gene expression variables (*FNIP2*, *FNIP1*, and *FLCN*), plus sex and age. Importantly, the inclusion of a FNIP2*rs2291007 interaction was significant and increased the optimism-corrected *R*^2^, so it was accepted in the final model. Figure 6A shows the estimated parameters of this model, and Fig. 6B displays the result of the bootstrap validation. We found that increased *FNIP2* expression associates with a decreased BMI (*beta*=−3.08, 95% CI (−3.92, −2.25), *p-*value= 2.12×10^−15^). Conversely, the beta parameter for the FNIP2*rs2291007 interaction is positive (*beta*=0.636, 95% CI (0.35, 1.52), *p-*value= 0.03), which means that the inclusion of a T variant results in a significantly less negative slope, and thus to a less dramatic decrease of BMI with higher *FNIP2* expression values. The inclusion of miR181-5p expression did not result in an improved model nor did the interactions between gender and rs2291007 (gender*rs2291007) or between gender and *FNIP2* gene expression (gender*FNIP2), neither the use of a codominant or dominant assumption for the SNP.

The variable importance plot for the multifactorial model of BMI prediction (Fig. 6C) establishes that the most important variable is the *FNIP2* expression, followed by age, *FLCN*, *FNIP1*, and the FNIP2*rs2291007 interaction. Collectively, these results provide evidence that rs2291007-*FNIP2*-Folliculin complex could modulate overweight and obesity.

## Discussion

Obesity and overweight are prevalent in developed countries and predispose to several comorbidities and diseases, such as most types of cancer. Recently, the impact that obesity has on the immune system has acquired a novel dimension of importance for the quality of life of the global population [5] because of the positive association between weight and adverse effects of SARS-CoV2 infection. Obesity is preventable, but causes and consequences must be deeply understood to design efficient preventive measures and tools [23]. Many parallel efforts have been undertaken to understand the genetic bases of this disease, including several GWAS studies that have opened a window of knowledge with the early identification of the *FTO* gene as the first genetic determinant of human body weight [8, 9]. While additional studies have further contributed to pinpointing genetic bases for the predisposition to obesity, this knowledge can explain so far approximately 20% of genetic predisposition. We restricted a genetic window of analysis to components of a metabolically relevant pathway, the mTOR pathway (Additional file 1: Fig. S1), due to its involvement in the sensing of energy, nutrients, and stress, as well as growth factors, and its deregulation in the obesity state [24].

In this work, our analysis of common genetic variations in this selected set of genes in almost 800 individuals of European origin revealed an association of rs2291007 in *FNIP2* with BMI and phenotypic characteristics of the obese state (Fig. 1A). The polymorphism rs2291007 is located in the 3′UTR of the *FNIP2* locus, and it has not been previously associated in the literature with overweight or obesity. Other variations in the *FNIP2* gene have been associated in GWAS studies with several conditions (depression, intelligence; schizophrenia, smoking initiation; coffee consumption measurement) but none related to BMI or metabolic alterations (https://www.ebi.ac.uk/gwas/genes/FNIP2). Notably, germ-line mutations in other members of the folliculin complex, *FLCN* gene, are associated with Birt-Hogg-Dube syndrome, which is characterized by fibrofolliculomas, renal tumors, lung cysts, and pneumothorax [25]. For first time, this study describes that the minor allele T of rs2291007 in the *FNIP2* gene is associated with metabolic and obesity-related phenotypes (elevated fat mass, visceral fatness, weight, BMI, and waist and hip circumferences) and with decreased muscle mass in healthy European individuals (Fig. 1A,B).

The aforementioned SNP is located within a conserved microRNA binding sequence. Typically, microRNAs posttranscriptionally regulate mRNA expression by binding to the 3′UTR of target mRNAs [26]. Because miRNA binding sites are constrained by secondary stabilizing interactions with the target mRNAs, SNPs located in 3′UTR sequences can result in differential miRNA binding, as they may either abolish or create a microRNA target, thus affecting mRNA levels [27]. We have shown, in cultured cells, that miR-181b-5p binds to the rs2291007 region and abolishes the expression of *FNIP2* through binding selectively to the T allele (Fig. 2). Consistently, T carriers of rs2291007 showed diminished *FNIP2* expression in peripheral blood, and importantly, its expression strongly associates with decreased obesity-related metabolic parameters (Fig. 3).

The conservation of the 3′UTR sequence of the *FNIP2* SNP across mammals allowed us to interrogate the functional effect of this T-to-C human-specific nucleotide change in mice by knocking-in the rs2291007 C variant in the mouse genome. The lack of commercial antibodies for mouse Fnip2 protein precluded its quantification, but mRNA levels of *Fnip2*^C^ and *Fnip2*^T^ could be ascertained. While differences between the two variants were not detected in steady-state conditions, increased levels of the *Fnip2*^C^ mRNA were seen upon acute inhibition of RNA pol II activity with Actinomycin D, consistently with the prediction. Future work may uncover the physiological setting in which mRNA stability yields a difference in Fnip2 protein levels, and thus, on the control of mTORC1 activity, which is seen affected in GSEA of samples from *Fnip2*^C/C^ and *Fnip2*^T/T^ mice (Fig. 5E, Additional file 1: S5E) but not acutely in cells derived from them (Fig. 4D). Alternatively, *Fnip2* levels may be more strikingly affecting other functions reported to be controlled by *FNIPs* [28], or other, still unknown metabolic roles.

Regardless of the timing and molecular cues that provide a functional explanation for the differences seen in carriers of the *FNIP2* T versus C SNP in healthy volunteers, there is a remarkable correlation between the observations in human and mouse: *Fnip2*^C/C^ mice show the same decrease in fat content as that observed in volunteers, a difference that is more prominent in female mice at all ages. RNA sequencing analyses revealed that the transcriptome of the WAT is largely affected by the presence of the *Fnip2* variant, with more than 4000 genes significantly different, in contrast to less than a dozen genes differentially expressed in liver samples. Moreover, samples from both livers and WAT from *Fnip2*^T/T^ mice show a dramatic and consistent enrichment in adipogenesis and fatty acid metabolism-related gene sets (Fig. 5E, F; Additional file 1: Fig. S5E and S5F), consistently with the association between the expression of the *FNIP2* T allele and increased adiposity found in both species. In addition, the same negative correlation between the levels of human *FNIP2* mRNA and body weight is seen when analyzing *Fnip2* mRNA levels and mouse weight. This whole recapitulation is more remarkable when considering that rs2291007 has linkage disequilibrium with additional variants in the genomic region of *FNIP2*, which are not modeled in these novel knock-in mice, thus suggesting that the T-to-C change is the main modulator of human leanness within the *FNIP2* locus.

The identification of relevant biomarkers linked to obesity and its comorbidities is an unsolved challenge [29]. PBMCs can serve as a source of molecular biomarkers for diverse metabolic alterations: PBMCs are easily sampled and mirror changes in the expression of metabolic genes in internal organs, thus providing accessible information on changes in the early obese state [30]. Thus, based on our results, we designed a multifactorial model to predict BMI in a healthy population. We propose a peripheral blood biomarker for BMI composed of *FNIP2*, *FLCN*, and *FNIP1* expression, plus age of the patient and the effect of rs2291007 (Fig. 6). Although our findings were highly consistent and bootstrap validation was implemented, further studies in an independent cohort should be performed in order to validate the multifactorial model for BMI prediction. Prediction of overweight and obesity can be of paramount importance for early therapeutic interventions focused on obesity-related comorbidities.

Collectively, our work supports a critical role for *FNIP2* in the control of human leanness.

## Conclusions

- We found an association of rs2291007 minor allele T in the *FNIP2* gene with metabolic and obesity-related phenotypes and with decreased muscle mass in healthy European individuals.
- T carriers of rs2291007 have decreased expression of *FNIP2* in peripheral blood, and importantly, *FNIP2* mRNA levels strongly associate with decreased obesity-related metabolic parameters.
- *Fnip2*^C/C^ knock-in mice replicate the decreased adiposity and increased leanness observed in human volunteers.
- Primary cells derived from *Fnip2*^C/C^ mice show increased mRNA stability.
- We propose a peripheral blood biomarker for BMI composed of *FNIP2* expression, age of the patient, and the effect of rs2291007 on *FNIP2* expression.
- Together, our works revealed a crucial role of *FNIP2* in the control of human leanness.

## Methods

### Subjects and sample structure

Genotyping was carried out on 790 volunteers belonging to the Platform for Clinical Trials in Nutrition and Health (GENYAL Platform) of IMDEA-Food Institute. Among them, RNA from 161 volunteers was used for gene expression analysis and microRNA studies were performed in 89 individuals. Volunteers, aged between 18 and 72 years, were recruited during the period 2012–2018 and did not suffer from any serious diseases. All contributors signed the consent participation form. These studies were approved by the IMDEA Food Research Ethics Committee and methodologies conformed to the standards set by the Declaration of Helsinki.

Relevant phenotypic information is summarized in Additional file 2: Table S1 [31–40]. Anthropometric magnitudes were measured under standardized methods. Height (cm) was assessed to the nearest 0.1 cm using a stadiometer (Leicester Biológica Tecnología Médica SL, Barcelona, Spain). Weight (kg), fat mass (%), and muscle mass (%) were estimated using bioelectrical impedance analysis (Body Composition Monitor BF511-OMRON HEALTHCARE, LT, Kyoto, Japan). Body mass index (BMI) was calculated using these estimates and as defined by the Quetelet Index (weight (kg)/height (m)^2^). The World Health Organization’s criteria (WHO) was employed to catalog the volunteers as normal weight when BMI < 25 kg/m^2^ and as overweight when BMI ≥ 25 kg/m^2^. Waist and hip circumference were measured with a flexible Dry 201 metal tape, with measuring range 0–150 cm and 1 mm of precision (Quirumed, Valencia, Spain). Blood sample extractions, biochemical determinations (lipid and glycemic profiles), and nutritional assessments (dietary and physical activity parameters) were collected following an overnight fasting as previously described [41–44].

### SNP genotyping

Genetic information was obtained from NCBI-dbSNP (https://www.ncbi.nlm.nih.gov/snp/), Ensembl (http://www.ensembl.org/index.html), and GWAS catalog (https://www.ebi.ac.uk/gwas/). Fifty-six SNPs in 25 genes of the mTOR pathway (Additional file 1: Fig. S1 and Additional file 2: Table S2) were selected.

Genomic DNA from peripheral blood was extracted using the QIAamp DNA Blood Mini Kit (Qiagen Sciences, Inc, Germantown, MD, USA). Genotyping was performed with QuantStudio 12 K Flex Real-Time PCR System (Life Technologies Inc., Carlsbad, CA, USA) using TaqMan OpenArray plates following the manufacturer’s instructions and results were analyzed using TaqMan Genotyper software.

### MicroRNA experiments

Two in silico tools were used for predicting miR181 structure and binding to rs2291007 region: TargetScanHuman (http://www.targetscan.org/vert_72/) for prediction of microRNA targets [19], and Sfold (https://sfold.wadsworth.org/cgi-bin/index.pl) for statistical folding of nucleic acids and studies of regulatory RNAs [20].

Sequences for *FNIP2* 3′UTR with rs2291007 C or T alleles were cloned into psiCheck2 by ATUM (Newark, California). HEK-293T cells were transfected using Lipofectamine 2000 (Life Technologies, Thermo Fisher Scientific, Waltham, MA, USA) according to the manufacturer’s recommendations. Each reaction contained 100 ng of either psiCheck2-3′UTR-*FNIP2*-rs2291007-C or psiCheck2-3′UTR-*FNIP2*-rs2291007-T and 30 nM of miR181b mimic miRNA (mirVana® miRNA mimic, Thermo Fisher Scientific, Waltham, MA, USA) [45]. Relative luciferase activity (Renilla luminescence/firefly luminescence) was determined after transfection using the Dual-Luciferase Reporter assay system (Promega, Madison, WI, USA) as surrogate for the translational repression of *FNIP2* upon binding to their 3′UTR of miR181b.

### mRNA expression analysis

For human peripheral blood samples, we used TRIzol method or RNeasy Mini Kit (Qiagen, Germantown, MD, USA) following the manufacturer’s conditions to obtain total RNA. Reverse transcription was performed with the High-Capacity cDNA Reverse Transcription kit (Thermo Fisher, Madrid, Spain), following the manufacturer’s instructions. RT-PCR reactions were performed as previously described [46] using QuantStudio 12 K Flex Real-Time PCR System (Life Technologies Inc., Carlsbad, CA, USA), with specific Taqman probes: *FNIP2* (Hs01574322_m1), *FLCN* (Hs00376065_m1), *FNIP1* (Hs00382846_m1), *GAPDH* (Hs02786624_g1). The 2^−ΔΔCt^ method was applied to calculate the relative gene expression. COSMIC Cell Lines Project (https://cancer.sanger.ac.uk/cell_lines) was used to download genotypes and gene expression values for *FNIP2* in 965 cell lines.

For mouse tissues and primary cells, RNA was extracted using TRIzol together with RNeasy Mini Kit (Qiagen, 74106) or Direct-zol RNA Miniprep (Zymo Research, R2051). Reverse transcription was performed using SuperScript IV VILO Master Mix (Invitrogen, 11756500). Quantitative real-time PCR was run in triplicates using GoTaq qPCR Master Mix (Promega, A6001) in a QuantStudio 6 Flex Real-Time PCR System thermocycler (Applied Biosystems). The 2^−ΔΔCt^ method was applied to calculate the relative gene expression, using *β-actin* as reference gene.

### microRNA expression analysis

Total RNA obtained from human peripheral blood samples and from mouse primary cells was retrotranscribed using TaqMan microRNA reverse transcription kit (Applied Biosystems, 4366596). Real-time PCR was run in triplicates using TaqMan Fast Advanced Master Mix (Applied Biosystems, 4444557) with specific TaqMan probes: hsa-miR-181b (4427975, MI0018778), U6 snRNA (4427975, NR_004394), mmu-miR-181b-5p (4440886, MI0000723), and snoRNA202 (4427975, AF357327).

### Mouse genome engineering

To engineer *Fnip2* rs2299007 C allele in mice, C57BL/6 mouse blastocysts were injected with Cas9, a single-guide (sg) RNA targeting the sequence of interest and a repair single-stranded oligonucleotide containing the intended mutations flanked by 70 bp of homology arms adjacent to the double-strand break site. Genotyping was performed by specific amplification followed by restriction fragment length polymorphism (RFLP) and/or Sanger sequencing.

sgRNA: GATAAGTGATATGAATGTAT

Repair single-stranded oligonucleotide:

CCGAGCAGAAGTGTCTCAGTGTCCTGTAATGACCTCTTCTAGCATGTTGCAGTTTTATATTTGTTAAGTTGATAAGTGATATGAACGTATACGCAATTGTGTATGTTTTCAAAAAGGACAATGAAAATTTAAAATGTAGCTTCCACACTTGTGCATAATTCCA

### Mouse experimentation

All animal procedures carried out at the CNIO were performed according to protocols approved by the CNIO-ISCIII Ethics Committee for Research and Animal Welfare (CEIyBA), under protocol number PROEX 015/18. Mice were housed under specific pathogen-free (SPF) conditions, at 22 °C and with 12-h dark/light cycles. Mice were fed with a standard chow diet (Harlan Teklad 2018). For fasting experiments, mice were placed in clean cages without access to food from 4 pm to 8 am.

### Animal imaging

For densitometry analysis, body composition (body weight, fat mass and lean muscle mass) was measured using dual-energy X-ray absorptiometry (DEXA) PIXImus, Mouse Densitometer (GE Lunar co, Madison, WI, USA), software version 1.46. During the measurements, mice were anesthetized with isoflurane. Quality control was performed using a calibrated phantom before imaging.

### Mouse embryonic fibroblasts

MEFs were isolated from E13.5 embryos using a protocol consisting of a chemical digestion with trypsin followed by mechanical disaggregation. For amino acid starvation experiments, cells were rinsed 3 times and placed in RPMI medium without amino acids (US Biological, R8999-04A) supplemented with 10% dialyzed serum. Amino acid stimulation was performed adding a cocktail containing the 20 amino acids (with concentration as in RPMI) directly to cell culture plates.

### Immunoblotting

Cells were rinsed once with ice-cold PBS and lysed in ice-cold protein lysis buffer containing 50 mM HEPES (pH 7.4), 40 mM NaCl, 2 mM EDTA, 1.5 mM sodium orthovanadate, 50 mM NaF, 10 mM pyrophosphate, 10 mM glycerophosphate, and 1% Triton X-100 and one tablet of complete protease inhibitors (Roche) per 25 ml. Cell lysates were cleared by centrifugation at maximum speed for 10 min. Protein content was measured from extracts with BCA Protein Assay. Protein extracts were denatured by adding sample buffer and boiling for 5 min, resolved by SDS-PAGE and analyzed by immunoblotting. Western blot analyses were performed according to standard procedures. Results were visualized using Odyssey Infrared Imaging System LI-COR Biosciences. The following primary antibodies were used: P-T389-S6K (Cell Signaling Technology, 9234), S6K (Cell Signaling Technology, 2708), TFEB (Bethyl Laboratories, A303-673A), and β-actin (Sigma-Aldrich, A1978).

### mRNA stability measurements

For mRNA stability measurements MEFs were treated with 5 μg/mL Actinomycin D for 2 and 4 h to stop transcription. RNA was extracted using Direct-zol RNA Miniprep (Zymo Research, R2051).

### Statistical analysis in human studies

Descriptive analysis was implemented for different continuous and categorical variables. Associations between gene expression variables were analyzed through Pearson correlation coefficients and tested through the corresponding test. Differences between gene expression in two groups were tested through Student’s *t* test, or through linear models with adjustment variables. SNPs were categorized by genotype (homozygote minor allele, heterozygote, and homozygote major allele). Deviations from Hardy-Weinberg equilibrium of genotype frequencies at individual loci were assessed using standard *χ*^2^ tests. Linkage disequilibrium was quantified through the *r*^2^ and D’ statistics.

Associations of the SNPs with numerical variables were modeled through linear regression on the SNP adjusted by sex and age. Associations with binary variables were modeled through logistic regression adjusted by sex and age. An additive model for the SNPs (effect in the homozygote minor allele is twice as much as that of the heterozygote) was considered by default, as in general it gave better fits, although in some cases a recessive or dominant model was considered.

A final multifactorial model to predict BMI was validated through bootstrap, using 2000 bootstrap samples, which allowed to correct the performance statistics (*R*^2^, MSE or Mean Square Error) by optimism in order to assess its predictive power and possible overfit.

This final model was obtained by sequentially adding SNP and gene expression variables to a basal model with only sex and age as predictor variables. A new variable was accepted if it was statistically significant and in addition produced a new model with an increased optimism-corrected *R*^2^, in order to prevent overfit. In the final model, the relative importance for the different predictor variables was estimated from the χ^2^- df (degrees of freedom) of the corresponding variables.

All tests were bilateral, with a significance level of 0.05. Ninety-five percent confidence intervals (95% CI) were used in parameter estimates (betas and odds ratios). Bonferroni method was applied for multiple-test correction of the *p*-values. All statistical analyses were performed using the R statistical software, version 3.6.1 (www.r-project.org).

### Statistical analysis in mouse studies

Statistical analyses were carried out with Prism 8 (GraphPad). Experiments including a second variable (e.g., time or nutritional status) were analyzed using 2-way ANOVA with Sidak’s multiple comparison post-test. Where appropriate, the area under the curve (AUC) was calculated. Unpaired Student’s *t* test was used for single comparisons. Associations between gene expression levels and body weight were modeled using a simple linear regression.

## Supplementary Information

Additional file 1: Figure S1. Components of the nutrient-mTORC1 pathway. Cartoon with the regulatory network of genes within the mTORC1 pathway. Figure S2 (related to main Fig. 1). Genetic associations. Figure S3 (related to main Fig. 3). Transcriptomic consequences of rs2291007. Figure S4 (related to main Fig. 4). A *Fnip2*^C^ Knock-in mouse to model *FNIP2* rs2291007. Figure S5 (related to main Fig. 5). Impact of the engineered T-to-C substitution on mouse adiposity. Figure S6. Un-cropped Western blots.

Additional file 2: Table S1. Phenotypic characteristics of healthy volunteers (Full set and population subsets). Table S2. List of SNPs, genes, location, type, and minor allele frequencies genotyped in this study. Table S3. Detailed information of SNPs associations with metabolic characteristics. Table S4. Associations of FNIP2, FNIP1, FLCN and miR-181b expression with metabolic phenotypes.

Additional file 3. Review history.

## Data Availability

The datasets used and/or analyzed during the current study are available from the corresponding author on reasonable request and in Gene Expression Omnibus database (GSE200620 [47] and GSE215158 [48]). Data of *FNIP2* mRNA levels were directly downloaded and used from Catalogue of Somatic Mutations in Cancer (COSMIC) cell lines [21].
