# Supplementary material for: Folliculin-interacting protein FNIP2 impacts on overweight and obesity through a polymorphism in a conserved 3′ untranslated region
Source: Genome Biol. 2022 Oct 31;23:230. doi: 10.1186/s13059-022-02798-5 (PMC9620695; doi:10.1186/s13059-022-02798-5)
Supplement: Supplementary file 1 — Additional file 1: Figure S1. Components of the nutrient-mTORC1 pathway. Cartoon with the regulatory network of genes within the mTORC1 pathway. Figure S2 (related to main Fig. 1). Genetic associations. Figure S3 (related to main Fig. 3). Transcriptomic consequences of rs2291007. Figure S4 (related to main Fig. 4). A Fnip2C Knock-in mouse to model FNIP2 rs2291007. Figure S5 (related to main Fig. 5). Impact of the engineered T-to-C substitution on mouse adiposity. Figure S6. Un-cropped Western blots. [file 13059_2022_2798_MOESM1_ESM.pptx]

## Slide 1
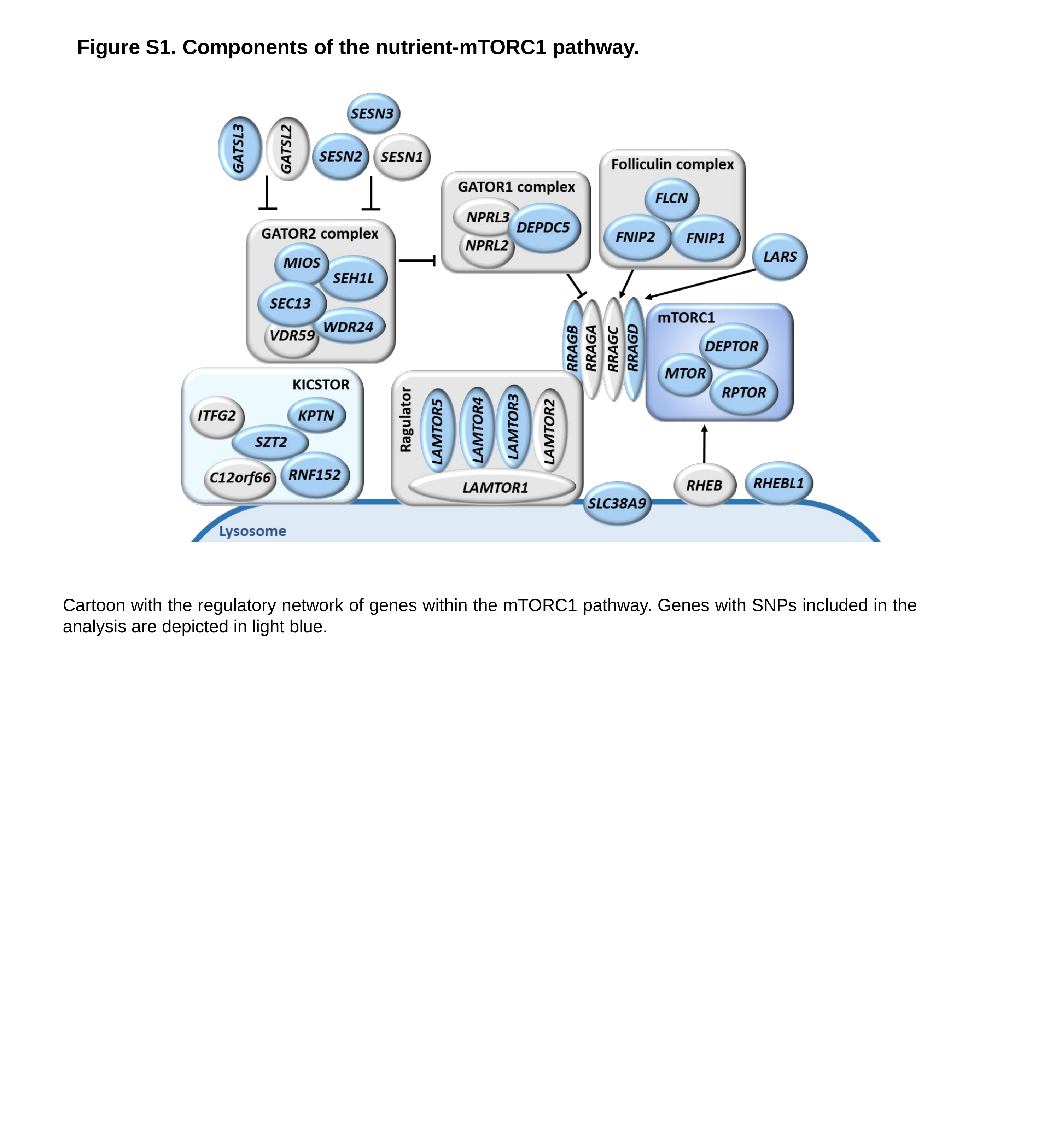

Figure S1. Components of the nutrient-mTORC1 pathway.
Cartoon with the regulatory network of genes within the mTORC1 pathway. Genes with SNPs included in the analysis are depicted in light blue.

## Slide 2
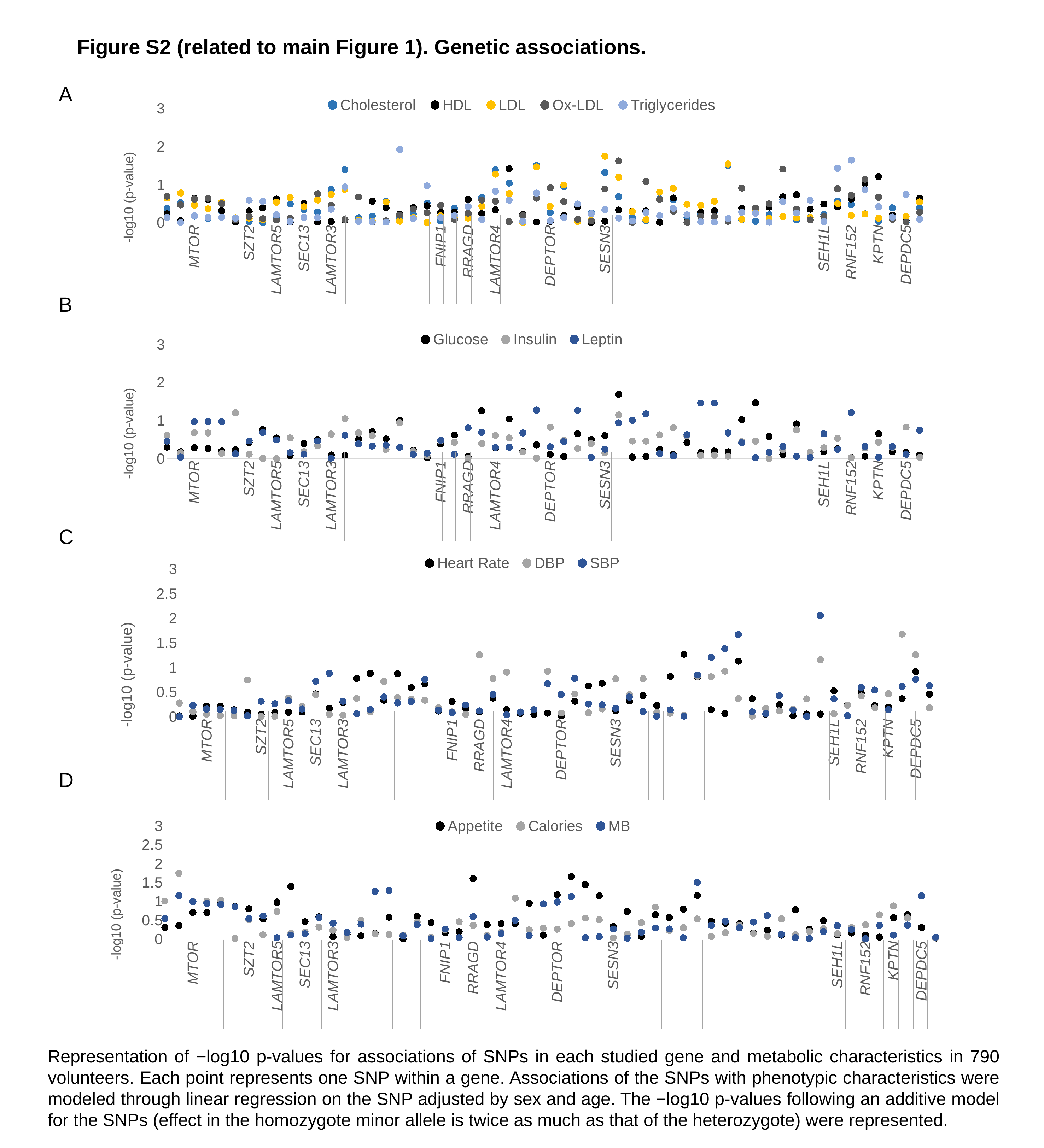

Figure S2 (related to main Figure 1). Genetic associations.
### Chart
| Category | Cholesterol | HDL | LDL | Ox-LDL | Triglycerides |
|---|---|---|---|---|---|
| | 0.3788237182249648 | 0.23642227553335465 | 0.6532559453951512 | 0.7020207558406376 | 0.1435127871313693 |
| | 0.5323918944163667 | 0.05615937359873888 | 0.7867479478036034 | 0.47391930819797007 | 0.012601900862982612 |
| MTOR | 0.17639537164484198 | 0.6456994376546403 | 0.46852108295774486 | 0.6274561992409297 | 0.18203819546800604 |
| | 0.1183871275216516 | 0.6085355881608968 | 0.3701828039814842 | 0.6472388082761692 | 0.15076490868527748 |
| | 0.2989503692708603 | 0.31713320433767545 | 0.5446982283429236 | 0.5077985486074603 | 0.15051916275601382 |
| | 0.05389176956309403 | 0.032031037121307684 | 0.13318119707395185 | 0.07597929952593241 | 0.1274358569093484 |
| SZT2 | 0.033295223342125445 | 0.312292720375181 | 0.12656313677796308 | 0.1688984354986406 | 0.5991167844516373 |
| | 0.00013030789173217684 | 0.3927594961682573 | 0.081759909778585 | 0.10896458468468917 | 0.5689580546641145 |
| LAMTOR5 | 0.15428198203334137 | 0.624885315307775 | 0.5398541825082498 | 0.07696333132921407 | 0.2109130849119713 |
| | 0.4952573637283121 | 0.018815862601645743 | 0.6699922991272408 | 0.12546020292898982 | 0.0387213209149569 |
| SEC13 | 0.3491099221436876 | 0.5191310763128322 | 0.42296801437396864 | 0.14966014541652078 | 0.145147637582166 |
| | 0.28617357561947543 | 0.018770512579999232 | 0.6018863082694975 | 0.7662426370344895 | 0.13882416448697127 |
| LAMTOR3 | 0.8751698505861408 | 0.0341400631929283 | 0.7503125721946985 | 0.45729903055188914 | 0.3602147870131798 |
| | 1.3968556273798178 | 0.07556563645676934 | 0.8823973083099157 | 0.08608023504853407 | 0.9461535731477474 |
| | 0.13465909437541604 | 0.06610796423578892 | 0.06833876031551878 | 0.6792307716613135 | 0.0399099320950805 |
| FNIP2 | 0.17140454362829763 | 0.5715412264844199 | 0.04268010314465918 | 0.022550772617658758 | 0.0329202658555029 |
| | 0.5730010412434628 | 0.397505931192719 | 0.5499049241283976 | 0.05286048235717089 | 0.019678413991243645 |
| SKP2 | 0.13560759484941132 | 0.22922160383085236 | 0.043975717719322883 | 0.20453710560962013 | 1.9303319030884043 |
| | 0.23980377054486604 | 0.3982658517398951 | 0.14660602254933422 | 0.3629107264696695 | 0.11565785235294108 |
| SLC38A9 | 0.5207126835238299 | 0.4528408786725825 | 0.008021209005416367 | 0.2670436304243754 | 0.9759250126925737 |
| FNIP1 | 0.049976093476673526 | 0.28025450747042335 | 0.18495382393536944 | 0.4613006204575932 | 0.14302956651750767 |
| LARS | 0.39104630072413715 | 0.28441444810680383 | 0.11815903167507256 | 0.09140776115243056 | 0.19003647828598602 |
| RRAGD | 0.12262865413022594 | 0.6165437034752469 | 0.1252283628157017 | 0.253599355508439 | 0.4295738216410274 |
| MIOS | 0.6681679555637513 | 0.24382048315619084 | 0.44081181099522454 | 0.6017126946425989 | 0.08793744441149766 |
| LAMTOR4 | 1.3942641061232535 | 0.34151161869098295 | 1.2820799741630065 | 0.5718652059712112 | 0.8303255659411931 |
| | 1.0476435226762093 | 1.4240043797967323 | 0.7698066211309544 | 0.03320145361663902 | 0.5979106494279033 |
| | 0.0343750328907572 | 0.2222108125651325 | 0.004671990922590423 | 0.1935486767527379 | 0.041722874452302425 |
| | 1.5114492834995557 | 0.01963297381522487 | 1.4714689393645886 | 0.648590248074561 | 0.7851561519523022 |
| DEPTOR | 0.2715650490257452 | 0.04148389657695891 | 0.43651891460558934 | 0.926648297613099 | 0.05305672930217456 |
| | 0.9507819773298184 | 0.1913839645730081 | 0.995248844408999 | 0.5586191150834886 | 0.14345235514325214 |
| | 0.08836306687055788 | 0.4255057317146725 | 0.03777276864991503 | 0.09253489323414378 | 0.49471432585586766 |
| | 0.26177455185749493 | 0.005946936412324747 | 0.06208109735221964 | 0.04905128560059965 | 0.23844801143581806 |
| SESN3 | 1.3240384503578306 | 0.04315950984076654 | 1.7554754884299162 | 0.8934691461776186 | 0.3508599358557811 |
| RHEBL1 | 0.6882461389442457 | 0.3398937782767559 | 1.2032869367191037 | 1.6294869104014074 | 0.12714475529518973 |
| | 0.1529211379342845 | 0.30697693207630594 | 0.2968806537639221 | 0.010238812281221806 | 0.04696554274964326 |
| WDR24 | 0.056901376994514724 | 0.31605286924848786 | 0.08544530148236637 | 1.0852338630741474 | 0.27384353382724513 |
| | 0.6362000545208907 | 0.015202742910707194 | 0.8068754016455384 | 0.6188849192901494 | 0.19158644859963192 |
| FLCN | 0.6088883862971974 | 0.6526699846830496 | 0.9108016331948511 | 0.30944953848964135 | 0.38216125228299663 |
| | 0.14972144748196284 | 0.017774008325325184 | 0.48704892002750944 | 0.009705553871561268 | 0.2143143317190987 |
| | 0.23195418589758326 | 0.2786016244784947 | 0.4626847268879903 | 0.17639537164484198 | 0.031330298279608 |
| | 0.30803489723263966 | 0.3175841383226415 | 0.5665502062384039 | 0.16500739626697014 | 0.020224067270314405 |
| | 1.5027938192960455 | 0.0502197916379994 | 1.5469876088785448 | 0.040433953362072346 | 0.1151480172540021 |
| | 0.08191721535781282 | 0.3796557002455067 | 0.09463593123310769 | 0.9175736991392281 | 0.2833290244398644 |
| RPTOR | 0.03720463014276677 | 0.26584048675553323 | 0.32239304727950685 | 0.3887016377035712 | 0.25336580106242124 |
| | 0.21588218353707672 | 0.41782296231159116 | 0.11901500951324659 | 0.4967542285348873 | 0.017367005605150372 |
| | 0.6095948435199191 | 0.6836102489268046 | 0.16545214231905134 | 1.4145392704914994 | 0.558304864359283 |
| | 0.07706704344478832 | 0.7454519228910261 | 0.1329451995232984 | 0.35467599843770664 | 0.26114019792779974 |
| | 0.14917004015146906 | 0.3619102780154943 | 0.13912320359670222 | 0.07805354577058984 | 0.5949953349496306 |
| SEH1L | 0.21745571598998992 | 0.4934949675951279 | 0.10023419805561389 | 0.1603962705291631 | 0.0293742233117055 |
| | 0.5681539543012746 | 0.4268962168360089 | 0.5069604116823487 | 0.8982529260536337 | 1.4381833356810425 |
| RNF152 | 0.4799096718871575 | 0.6272720591144045 | 0.19688444510997305 | 0.7276941555979136 | 1.6540384581868588 |
| | 0.236497134532403 | 1.0271956776634223 | 0.2347040703019434 | 1.1483747968466909 | 0.870310107800699 |
| KPTN | 0.038483988551051014 | 1.2189630613788682 | 0.12124847982699792 | 0.6757175447023074 | 0.4308602745275405 |
| GATSL3 | 0.39523411529611263 | 0.205581669125859 | 0.1449656833241159 | 0.09264239186882715 | 0.15273589822923547 |
| DEPDC5 | 0.020679302617975567 | 0.062030997048547164 | 0.17146899335489862 | 0.02108942282442832 | 0.7495799976911061 |
| RRAGB | 0.3949105381184196 | 0.6511112769285621 | 0.5509846836522136 | 0.28091742609851406 | 0.09415017335768122 |A
B
### Chart
| Category | Glucose | Insulin | Leptin |
|---|---|---|---|
| | 0.30715308072277 | 0.6161846340195687 | 0.4652126413705083 |
| | 0.18628560811885517 | 0.15664262156204442 | 0.04383156952463668 |
| MTOR | 0.29473513768259585 | 0.6869768896767618 | 0.9759250126925737 |
| | 0.27441502772930565 | 0.679853713888946 | 0.9759250126925737 |
| | 0.20432849405397824 | 0.14188406780993384 | 0.9759250126925737 |
| | 0.23995467203418938 | 1.212539525481585 | 0.13388996015562343 |
| SZT2 | 0.42782568638694046 | 0.12245589228405598 | 0.4682656907234498 |
| | 0.7677664788852664 | 0.009883233932095567 | 0.6912222263352789 |
| LAMTOR5 | 0.5469876088785448 | 0.007446482167864384 | 0.4978461071286392 |
| | 0.08444188458847955 | 0.5485205948751382 | 0.15889091553184612 |
| SEC13 | 0.4016472901307161 | 0.18495382393536944 | 0.12418661116024242 |
| | 0.5041782466140943 | 0.3422750457948918 | 0.46928816201834306 |
| LAMTOR3 | 0.09935981601739968 | 0.6491707264170322 | 0.01890657685407023 |
| | 0.0959339480854972 | 1.052615298315259 | 0.6228759576535439 |
| | 0.5198492747267196 | 0.679853713888946 | 0.39093945006991304 |
| FNIP2 | 0.7088532382681144 | 0.6073030467403343 | 0.335734199852325 |
| | 0.5231683714877393 | 0.24504041227829013 | 0.35931984672233486 |
| SKP2 | 1.007623240201197 | 0.9503943874050268 | 0.3019864960608183 |
| | 0.22388020094701225 | 0.20093528064899183 | 0.11810202642698872 |
| SLC38A9 | 0.02507401391023757 | 0.07293775650699992 | 0.15064201833870106 |
| FNIP1 | 0.38647529714634754 | 0.4737899961583357 | 0.4879830305038733 |
| LARS | 0.6263525277907822 | 0.43320908761840826 | 0.11798803837334138 |
| RRAGD | 0.0576446502923232 | 0.014349026309050977 | 0.8133261325002549 |
| MIOS | 1.2665619729089388 | 0.401428336517859 | 0.6965879294032581 |
| LAMTOR4 | 0.28441444810680383 | 0.6174426780912142 | 0.3022477258322454 |
| | 1.0448897690294483 | 0.54591772926891 | 0.30495634117870585 |
| | 0.19784224681303847 | 0.18164212204164523 | 0.6800615600196914 |
| | 0.3653212478213177 | 0.017502630802287533 | 1.2810833139851387 |
| DEPTOR | 0.11560117444331373 | 0.8279811905754435 | 0.3161427945996536 |
| | 0.05739675115784342 | 0.4897231555826451 | 0.4508740732418889 |
| | 0.6619421245802438 | 0.2698639960033222 | 1.2734357838377552 |
| | 0.5077985486074603 | 0.40120949323688493 | 0.03777276864991503 |
| SESN3 | 0.6062493596519196 | 0.15409616110121746 | 0.24995468798823245 |
| RHEBL1 | 1.6957249495228717 | 1.153044674980176 | 0.9465373950745447 |
| | 0.04421607108308855 | 0.467882883751196 | 1.0109063073896745 |
| WDR24 | 0.05898575629443027 | 0.46419970917510234 | 1.1809169242562974 |
| | 0.247260306064672 | 0.6304131092636557 | 0.1344222925800709 |
| FLCN | 0.11243895906999096 | 0.8164454663811384 | 0.0754622822245102 |
| | 0.4294570601181025 | 0.6004993386853894 | 0.6315271615596382 |
| | 0.16190686155440168 | 0.0893755951107988 | 1.4649591867488394 |
| | 0.20307892466983124 | 0.0893755951107988 | 1.4649591867488394 |
| | 0.18322858766665354 | 0.0660068361687577 | 0.6777807052660807 |
| | 1.0318440628500296 | 0.4509967379742122 | 0.42136079003192767 |
| RPTOR | 1.4721124340472953 | 0.4631891340084585 | 0.027750864037404305 |
| | 0.5853608532629909 | 0.009084245493915564 | 0.17179138553205459 |
| | 0.11486503393656611 | 0.23232477597203996 | 0.32882715728491674 |
| | 0.9168558568569477 | 0.7627076624325413 | 0.06278310913729455 |
| | 0.16608828492862157 | 0.17457388223217687 | 0.035316185023959334 |
| SEH1L | 0.1836924005680602 | 0.29081487044975457 | 0.6548223834572959 |
| | 0.26760624017703144 | 0.5331323796458906 | 0.2426799034533277 |
| RNF152 | 0.03376437590141819 | 0.027797161620935564 | 1.2163822348092512 |
| | 0.0637879556797512 | 0.2850000325879575 | 0.3268868957617663 |
| KPTN | 0.6589613683224771 | 0.4358075393738022 | 0.04038628892895605 |
| GATSL3 | 0.18608557995139652 | 0.2982593675627875 | 0.3252306859845737 |
| DEPDC5 | 0.16513442009996201 | 0.8317972531573691 | 0.1183871275216516 |
| RRAGB | 0.09017663034908802 | 0.03630659476198122 | 0.749092300299144 |C
### Chart
| Category | Heart Rate | DBP | SBP |
|---|---|---|---|
| | 0.022825323979812625 | 0.28158135817034435 | 0.005330978174470584 |
| | 0.01385553528947943 | 0.10684878687013409 | 0.2358238676096693 |
| MTOR | 0.2204756566752101 | 0.05719853368205958 | 0.16146572948813143 |
| | 0.22105327203138328 | 0.03077052013735692 | 0.15527437202677405 |
| | 0.14545106418704923 | 0.02410886359820726 | 0.1321202165416204 |
| | 0.09189045960744782 | 0.7525177393229456 | 0.025212067786442156 |
| SZT2 | 0.05714899344566259 | 0.008153946355103294 | 0.31866829403083424 |
| | 0.08862929288386205 | 0.015517693597737256 | 0.26776711977950224 |
| LAMTOR5 | 0.09517630019905587 | 0.384049948343599 | 0.32679471822095474 |
| | 0.09914149529800836 | 0.2201150368073558 | 0.1592667653881933 |
| SEC13 | 0.467882883751196 | 0.45481663178459386 | 0.7237680420781664 |
| | 0.1763301867318636 | 0.05139145012356367 | 0.8860566476931632 |
| LAMTOR3 | 0.2926001688667513 | 0.03658998431977123 | 0.3205721033878811 |
| | 0.7838340977140069 | 0.37499639898513654 | 0.06363708040225555 |
| | 0.8840568230609449 | 0.10612732120491858 | 0.15273589822923547 |
| FNIP2 | 0.3390397082239164 | 0.7223907856959089 | 0.40340290437353976 |
| | 0.8794260687941501 | 0.39480273261162224 | 0.2839131462251679 |
| SKP2 | 0.595166283380062 | 0.36602684421032644 | 0.3136318965269638 |
| | 0.6681679555637513 | 0.3383765907707699 | 0.7639668528823641 |
| SLC38A9 | 0.11964380058076375 | 0.18355983204386134 | 0.13912320359670222 |
| FNIP1 | 0.31479586552898536 | 0.10452220372428538 | 0.08846953769283745 |
| LARS | 0.168194191325609 | 0.052713355322201574 | 0.24290777988106738 |
| RRAGD | 0.10963487855187583 | 1.263444152837364 | 0.12401322857151198 |
| MIOS | 0.38216125228299663 | 0.7827793443554812 | 0.45173345482925475 |
| LAMTOR4 | 0.15347733158371318 | 0.9072793553159009 | 0.044456557540257034 |
| | 0.07063217075990037 | 0.10182351650232342 | 0.09598811640261179 |
| | 0.05188057561946393 | 0.15033494452126722 | 0.14733705565543073 |
| | 0.07768983678560405 | 0.9273825234547635 | 0.6755117666923436 |
| DEPTOR | 0.022276394711152253 | 0.07904229404455074 | 0.4568014143623533 |
| | 0.31984585826562695 | 0.4655931008621229 | 0.7840981867959684 |
| | 0.6311554931741786 | 0.08523386307414751 | 0.2640818834687029 |
| | 0.6829818989518885 | 0.16367588429324828 | 0.2458046118101616 |
| SESN3 | 0.12598229613781375 | 0.7736579128363693 | 0.17373078060627378 |
| RHEBL1 | 0.3215726775661329 | 0.44891613481421966 | 0.40472428819790063 |
| | 0.43568909003939726 | 0.7749493038619513 | 0.10957898119908573 |
| WDR24 | 0.23262147588581947 | 0.08846953769283745 | 0.014124642691606345 |
| | 0.8213107602244102 | 0.07494587968815734 | 0.14429916916456276 |
| FLCN | 1.2719710455794815 | 0.02909550184621629 | 0.019269623464054652 |
| | 0.8173000966639574 | 0.8227521637443765 | 0.8538719643217619 |
| | 0.14636261804140574 | 0.8164454663811384 | 1.2111955069553517 |
| | 0.06671522765130518 | 0.9273825234547635 | 1.3836295277087305 |
| | 1.1322968667299023 | 0.37592426885431734 | 1.6755117666923436 |
| | 0.3698773571406879 | 0.01745741769705663 | 0.10242788617426937 |
| RPTOR | 0.058091225634400535 | 0.17334220812413065 | 0.06757697362326119 |
| | 0.24841299491768976 | 0.1243022380197921 | 0.43215054942689324 |
| | 0.0236500209967266 | 0.15739076038943792 | 0.1412823851397083 |
| | 0.058091225634400535 | 0.36663255488299307 | 0.009394788576080552 |
| | 0.059732608553987994 | 1.159016162679622 | 2.060380921043302 |
| SEH1L | 0.5310621943345387 | 0.06509774167768603 | 0.3672391115205612 |
| | 0.2405588028663024 | 0.23822246249182186 | 0.0263642265825925 |
| RNF152 | 0.49498575991589294 | 0.42458120878563993 | 0.60223374387355 |
| | 0.23143589048642657 | 0.18164212204164523 | 0.546375926408549 |
| KPTN | 0.19873352951037984 | 0.47301493144000434 | 0.1493537648169335 |
| GATSL3 | 0.3709983807130083 | 1.6808939406902237 | 0.624702261782661 |
| DEPDC5 | 0.917933065714887 | 1.2614572590712148 | 0.7647241233129476 |
| RRAGB | 0.4633153273790699 | 0.18203819546800604 | 0.6399749108106025 |D
### Chart
| Category | Appetite | Calories | MB |
|---|---|---|---|
| | 0.3074174377250909 | 1.0120657347678415 | 0.5421181032660077 |
| | 0.36361241418684337 | 1.7510463845042925 | 1.1585153906646068 |
| MTOR | 0.7090754406172457 | 0.9974020192800914 | 0.9948194874962197 |
| | 0.7090754406172457 | 1.0080212090054164 | 0.9535048356652917 |
| | 1.005814871797683 | 1.0288169591438385 | 0.9197343726601552 |
| | 0.8601209135987634 | 0.02756572304263488 | 0.8544928285903374 |
| SZT2 | 0.8082696066371436 | 0.5145625189236986 | 0.5468346074741428 |
| | 0.5356595153723327 | 0.11645212073195632 | 0.6176226965318863 |
| LAMTOR5 | 0.9842212436109591 | 0.7330630888408269 | 0.038341651362284734 |
| | 1.4009907601766565 | 0.15857795559764062 | 0.11036219959333225 |
| SEC13 | 0.46243274284732483 | 0.1951112553776086 | 0.1447231961699082 |
| | 0.5914208745913325 | 0.3239468753481285 | 0.5704091977766984 |
| LAMTOR3 | 0.07180905196124303 | 0.23314118897858238 | 0.42782568638694046 |
| | 0.12895273869450058 | 0.052370252615645485 | 0.17953580942231595 |
| | 0.08735289378168283 | 0.4993519366280881 | 0.3992448503603819 |
| FNIP2 | 0.15776531365276036 | 0.14363367634075203 | 1.2716462179787715 |
| | 0.5855280503706973 | 0.12320502379929942 | 1.292004253577071 |
| SKP2 | 0.01264661126420635 | 0.10751582063531237 | 0.08958906008531183 |
| | 0.6074789100680678 | 0.4585455712524111 | 0.3832095136702842 |
| SLC38A9 | 0.43997375108710773 | 0.054186873412661526 | 0.01202108412451794 |
| FNIP1 | 0.16979640107429586 | 0.25484710492309964 | 0.27107835362714067 |
| LARS | 0.20128031481499373 | 0.4613006204575932 | 0.040433953362072346 |
| RRAGD | 1.6078308505102639 | 0.3687592197644908 | 0.5989442742281562 |
| MIOS | 0.3891269996199483 | 0.09903237608087571 | 0.05873709068105023 |
| LAMTOR4 | 0.4130752918551797 | 0.18236853280948462 | 0.15131834597604962 |
| | 0.4171415377755007 | 1.0908720580107392 | 0.5032086842999576 |
| | 0.9558523791212772 | 0.24687675531828737 | 0.09767069414168125 |
| | 0.10352889952072274 | 0.29387989029729644 | 0.9374180157718369 |
| DEPTOR | 1.1788796762231764 | 0.2679280590001334 | 0.989700043360188 |
| | 1.6591594501876685 | 0.41206465136364395 | 1.1365581713862913 |
| | 1.4517334548292546 | 0.5611413405794381 | 0.04229695845116863 |
| | 1.1505805862031007 | 0.5175552080817347 | 0.06469430971007481 |
| SESN3 | 0.3387553910406665 | 0.03569321769606352 | 0.26970233790285036 |
| RHEBL1 | 0.7363639314118918 | 0.1329451995232984 | 0.029049065654575875 |
| | 0.06869471857832676 | 0.4362816600343223 | 0.18675269910239498 |
| WDR24 | 0.6526699846830496 | 0.8504729862456522 | 0.297224922098956 |
| | 0.5780671867214916 | 0.23202827861838118 | 0.2714838952402333 |
| FLCN | 0.7953374882517811 | 0.30451832350980257 | 0.041531681633056323 |
| | 1.1598939055432422 | 0.5361070110140927 | 1.5075189898711234 |
| | 0.4754740633736243 | 0.07468790850035051 | 0.3687592197644908 |
| | 0.42067379624474494 | 0.1767866867173325 | 0.47573373123302126 |
| | 0.40726823360603776 | 0.37079434289769625 | 0.3040807471686 |
| | 0.16222223044626669 | 0.15273589822923547 | 0.45456917053464874 |
| RPTOR | 0.24139085734025628 | 0.0753072969798142 | 0.6293019074244232 |
| | 0.10773839308446484 | 0.538651566352017 | 0.13024040521758964 |
| | 0.7848914189469068 | 0.12193801870998745 | 0.040433953362072346 |
| | 0.2632848663943889 | 0.20985563495709944 | 0.019360432556262814 |
| | 0.4964816872759253 | 0.2786841193941009 | 0.20321758829869221 |
| SEH1L | 0.122110574628516 | 0.15027355580367213 | 0.3601152580836957 |
| | 0.16399254087446855 | 0.3105135516357521 | 0.2577463300934063 |
| RNF152 | 0.10634918301403641 | 0.3863695650747594 | 0.018090829909207715 |
| | 0.05551732784983137 | 0.64762450499948 | 0.36774522331528636 |
| KPTN | 0.5713793273280611 | 0.8830603534492443 | 0.10963487855187583 |
| GATSL3 | 0.6493643917410457 | 0.5712174885030455 | 0.3744817710283624 |
| DEPDC5 | 0.3085648478559379 | 1.1534773315837132 | 1.150642018338701 |
| RRAGB | 0.03273290844021437 | 0.0335297362707156 | 0.052125345102301816 |Representation of −log10 p-values for associations of SNPs in each studied gene and metabolic characteristics in 790 volunteers. Each point represents one SNP within a gene. Associations of the SNPs with phenotypic characteristics were modeled through linear regression on the SNP adjusted by sex and age. The −log10 p-values following an additive model for the SNPs (effect in the homozygote minor allele is twice as much as that of the heterozygote) were represented.

## Slide 3
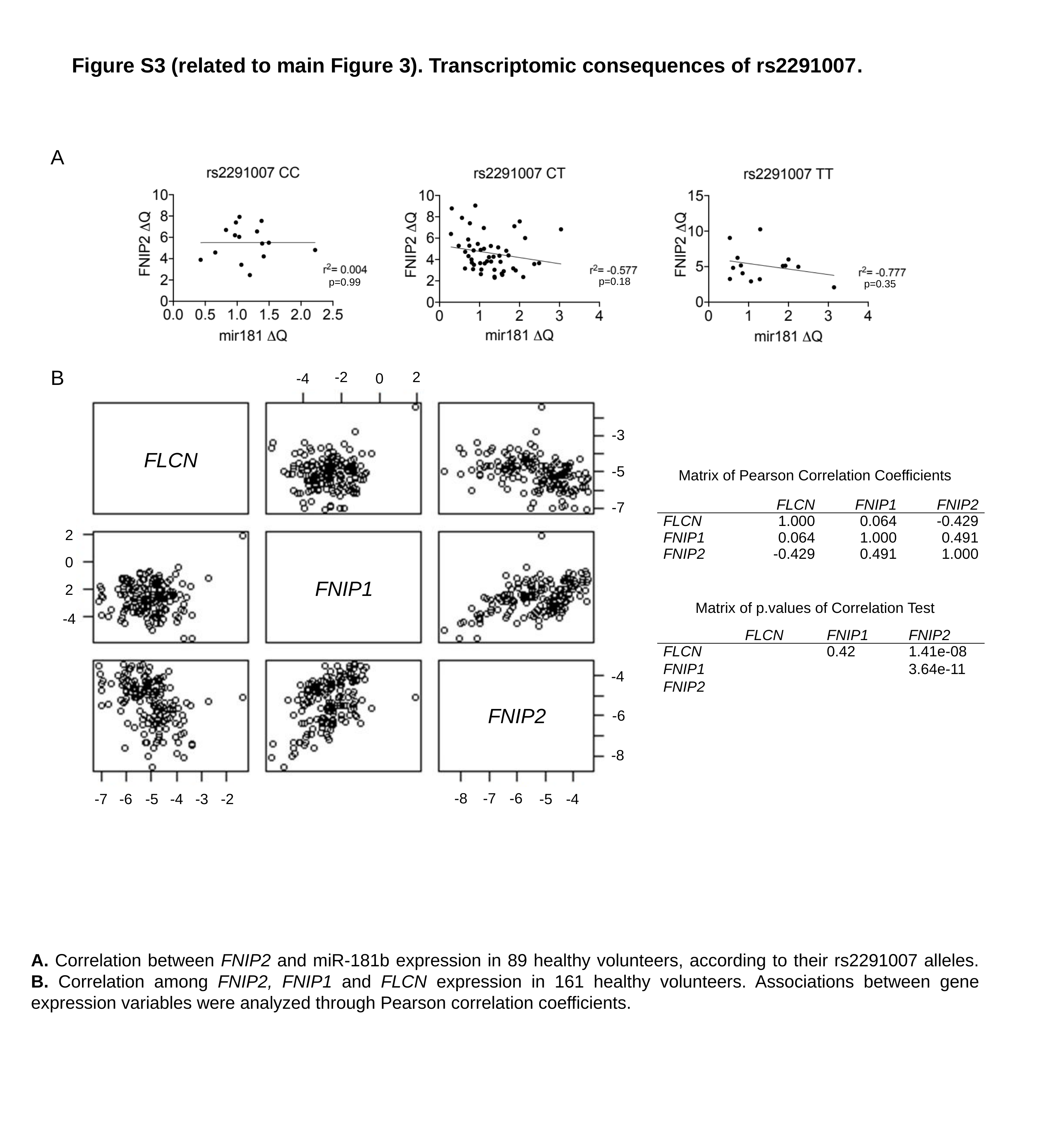

Figure S3 (related to main Figure 3). Transcriptomic consequences of rs2291007.
A
p=0.18
p=0.99
p=0.35
B
-2
2
-4
0
FLCN
-3
-5
-7
2
FNIP1
0
2
-4
-4
-6
-8
FNIP2
-8
-6
-7
-5
-4
-7
-6
-5
-4
-3
-2
Matrix of Pearson Correlation Coefficients
| | FLCN | FNIP1 | FNIP2 |
| --- | --- | --- | --- |
| FLCN | 1.000 | 0.064 | -0.429 |
| FNIP1 | 0.064 | 1.000 | 0.491 |
| FNIP2 | -0.429 | 0.491 | 1.000 |
Matrix of p.values of Correlation Test
| | FLCN | FNIP1 | FNIP2 |
| --- | --- | --- | --- |
| FLCN | | 0.42 | 1.41e-08 |
| FNIP1 | | | 3.64e-11 |
| FNIP2 | | | |
A. Correlation between FNIP2 and miR-181b expression in 89 healthy volunteers, according to their rs2291007 alleles. B. Correlation among FNIP2, FNIP1 and FLCN expression in 161 healthy volunteers. Associations between gene expression variables were analyzed through Pearson correlation coefficients.

## Slide 4
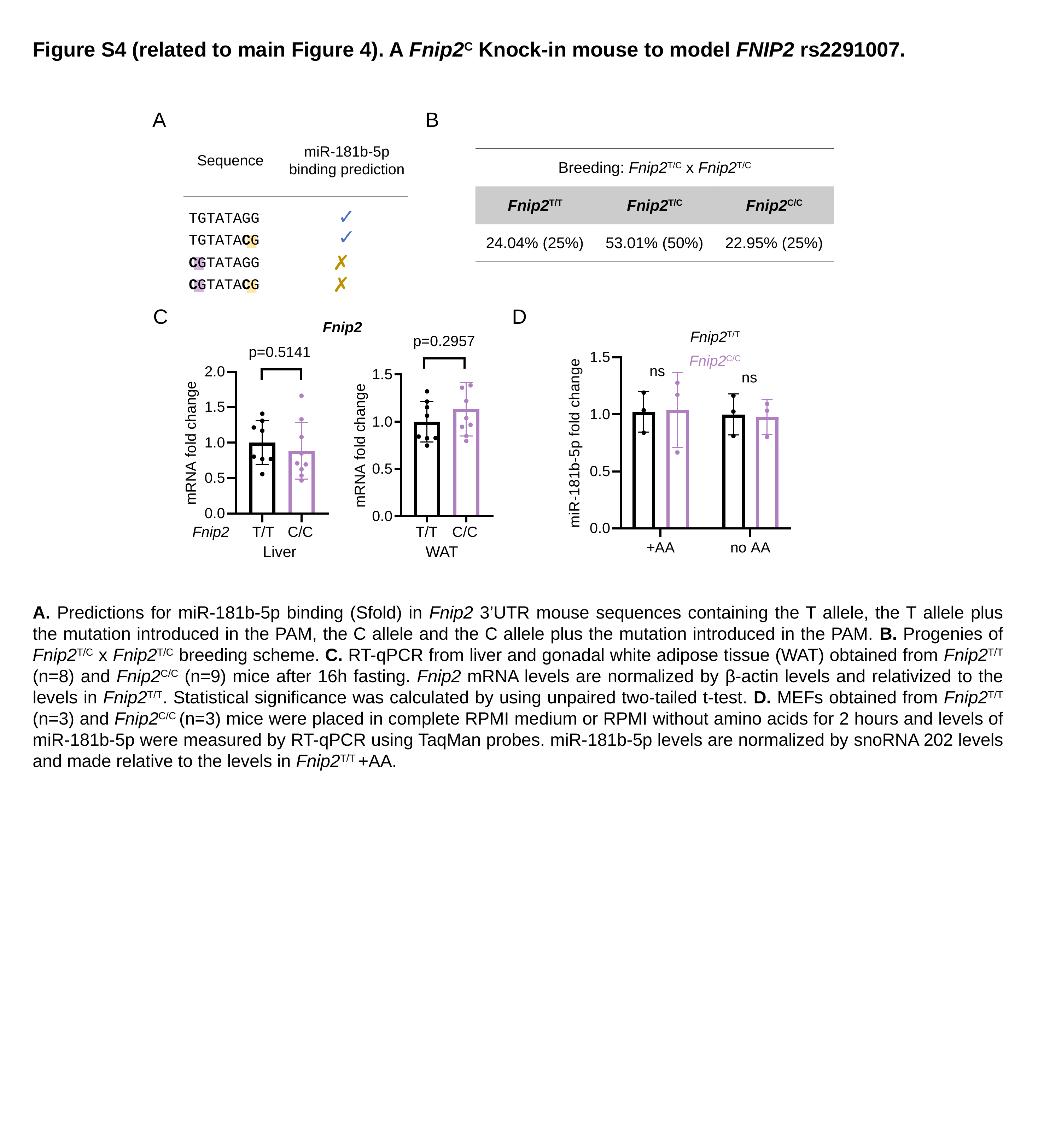

Figure S4 (related to main Figure 4). A Fnip2C Knock-in mouse to model FNIP2 rs2291007.
A
B
miR-181b-5p binding prediction
Sequence
| Breeding: Fnip2T/C x Fnip2T/C | | |
| --- | --- | --- |
| Fnip2T/T | Fnip2T/C | Fnip2C/C |
| 24.04% (25%) | 53.01% (50%) | 22.95% (25%) |
✓
TGTATAGG
✓
TGTATACG
✗
CGTATAGG
✗
CGTATACG
C
D
Fnip2
Fnip2T/T
Fnip2C/C
ns
ns
p=0.2957
p=0.5141
Fnip2
T/T
C/C
T/T
C/C
Liver
WAT
A. Predictions for miR-181b-5p binding (Sfold) in Fnip2 3’UTR mouse sequences containing the T allele, the T allele plus the mutation introduced in the PAM, the C allele and the C allele plus the mutation introduced in the PAM. B. Progenies of Fnip2T/C x Fnip2T/C breeding scheme. C. RT-qPCR from liver and gonadal white adipose tissue (WAT) obtained from Fnip2T/T (n=8) and Fnip2C/C (n=9) mice after 16h fasting. Fnip2 mRNA levels are normalized by β-actin levels and relativized to the levels in Fnip2T/T. Statistical significance was calculated by using unpaired two-tailed t-test. D. MEFs obtained from Fnip2T/T (n=3) and Fnip2C/C (n=3) mice were placed in complete RPMI medium or RPMI without amino acids for 2 hours and levels of miR-181b-5p were measured by RT-qPCR using TaqMan probes. miR-181b-5p levels are normalized by snoRNA 202 levels and made relative to the levels in Fnip2T/T +AA.

## Slide 5
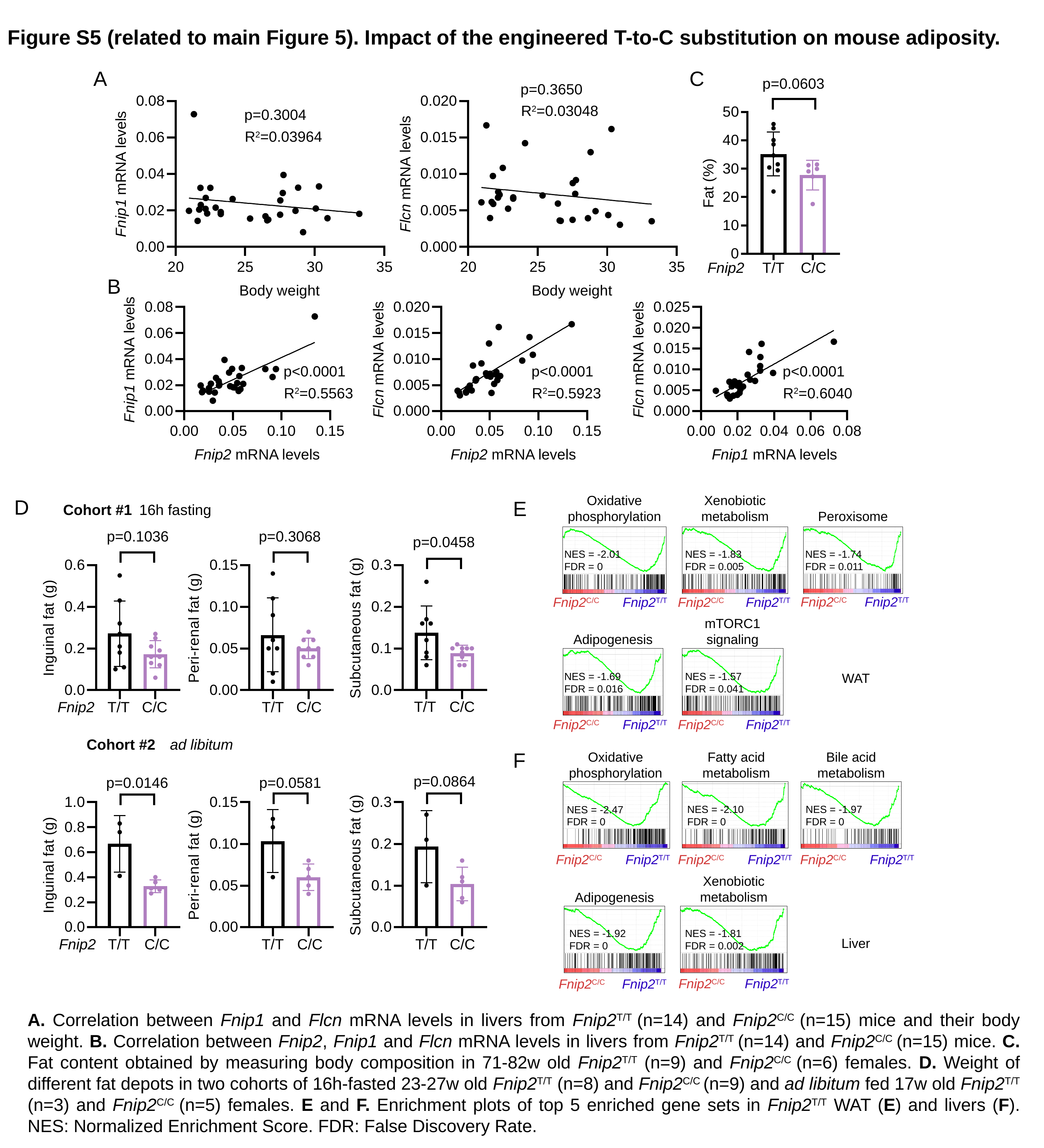

Figure S5 (related to main Figure 5). Impact of the engineered T-to-C substitution on mouse adiposity.
A
C
p=0.0603
p=0.3650
R2=0.03048
p=0.3004
R2=0.03964
Fnip2
T/T
C/C
B
Fnip2 mRNA levels
Fnip2 mRNA levels
Fnip1 mRNA levels
p<0.0001
p<0.0001
p<0.0001
R2=0.5563
R2=0.5923
R2=0.6040
Oxidative phosphorylation
Xenobiotic
metabolism
D
E
Cohort #1
16h fasting
Peroxisome
p=0.1036
Fnip2
T/T
C/C
p=0.3068
T/T
C/C
p=0.0458
T/T
C/C
NES = -2.01
FDR = 0
NES = -1.83
FDR = 0.005
NES = -1.74
FDR = 0.011
Fnip2C/C
Fnip2T/T
Fnip2C/C
Fnip2T/T
Fnip2C/C
Fnip2T/T
mTORC1
signaling
Adipogenesis
NES = -1.69
FDR = 0.016
NES = -1.57
FDR = 0.041
WAT
Fnip2C/C
Fnip2T/T
Fnip2C/C
Fnip2T/T
Cohort #2
ad libitum
F
Oxidative phosphorylation
Fatty acid
metabolism
Bile acid
metabolism
p=0.0864
T/T
C/C
p=0.0581
T/T
C/C
p=0.0146
Fnip2
T/T
C/C
NES = -2.10
FDR = 0
NES = -1.97
FDR = 0
NES = -2.47
FDR = 0
Fnip2C/C
Fnip2T/T
Fnip2C/C
Fnip2T/T
Fnip2C/C
Fnip2T/T
Xenobiotic
metabolism
Adipogenesis
NES = -1.81
FDR = 0.002
NES = -1.92
FDR = 0
Liver
Fnip2C/C
Fnip2T/T
Fnip2C/C
Fnip2T/T
A. Correlation between Fnip1 and Flcn mRNA levels in livers from Fnip2T/T (n=14) and Fnip2C/C (n=15) mice and their body weight. B. Correlation between Fnip2, Fnip1 and Flcn mRNA levels in livers from Fnip2T/T (n=14) and Fnip2C/C (n=15) mice. C. Fat content obtained by measuring body composition in 71-82w old Fnip2T/T (n=9) and Fnip2C/C (n=6) females. D. Weight of different fat depots in two cohorts of 16h-fasted 23-27w old Fnip2T/T (n=8) and Fnip2C/C (n=9) and ad libitum fed 17w old Fnip2T/T (n=3) and Fnip2C/C (n=5) females. E and F. Enrichment plots of top 5 enriched gene sets in Fnip2T/T WAT (E) and livers (F). NES: Normalized Enrichment Score. FDR: False Discovery Rate.

## Slide 6
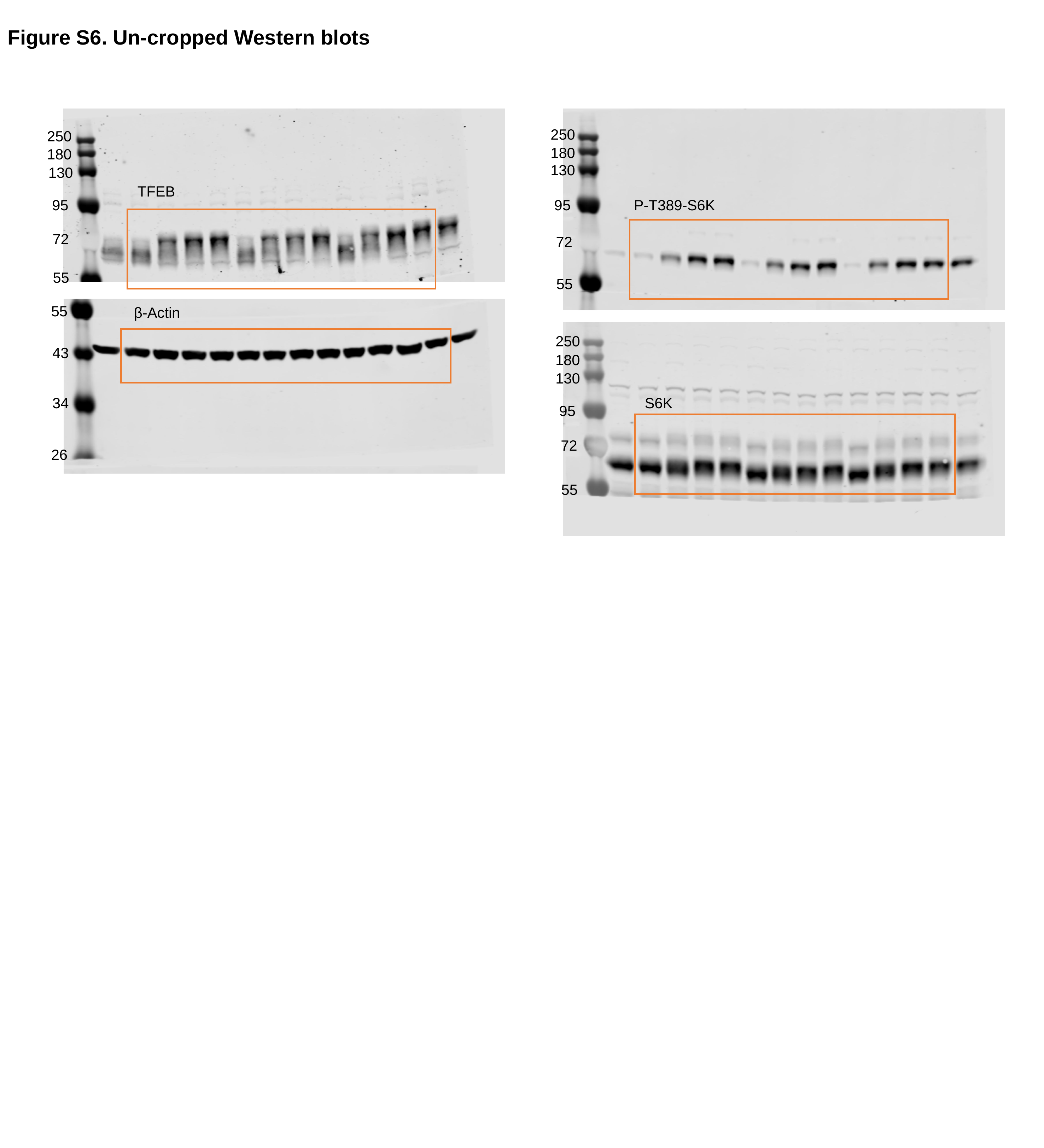

Figure S6. Un-cropped Western blots
250
250
180
180
130
130
TFEB
95
P-T389-S6K
95
72
72
55
55
55
β-Actin
250
43
180
130
S6K
34
95
72
26
55
