# Supplementary material for: Folliculin-interacting protein FNIP2 impacts on overweight and obesity through a polymorphism in a conserved 3′ untranslated region
Source: Genome Biol. 2022 Oct 31;23:230. doi: 10.1186/s13059-022-02798-5 (PMC9620695; doi:10.1186/s13059-022-02798-5)
Supplement: Supplementary file 3 — Additional file 3. Review history. [file 13059_2022_2798_MOESM3_ESM.docx]

Review History

**First round of review**

**Reviewer 1**

**Were you able to assess all statistics in the manuscript, including the appropriateness of statistical tests used?** No, I do not feel adequately qualified to assess the statistics.

**Comments to author:**

This study described in this manuscript addresses FNIP2 polymorphism in obesity-related phenotypes, based on the authors' observation that a single nucleotide polymorphism in FNIP2 is associated with obesity state in human. The data show that rs2291007, a T-to-C variant at the 3' UTR region of FNIP2 in increases FNIP2 mRNA stability. In human and a mouse model this increase in FNIP2 expression correlates with a decreased adiposity and increased leanness. This is an interesting study that supports a potential use of FNIP2 and FLCN levels in peripheral blood as a biomarker for body mass index prediction. It also bears an important implication for the function of the FLCN-FNIP complex controlling adipogenesis and body fat deposition. The manuscript is well-organized, and data clearly presented, nevertheless, there are a couples of issues need clarification.

1. While data show that miR-181b-5p binds to the T allele of FNIP2, there is no evidence that the miRNA contributes to obesity-related phenotypes in human and the animal model. The authors should include a discussion about the potential role of the miRNA in metabolic phenotypes.

2. Are there associations of FLCN and FNIP2 levels with aging or sex?

3. Figure 2C, does lower half of the figure depict miR-181a -5p binding or is a typo?

**Reviewer 2**

**Were you able to assess all statistics in the manuscript, including the appropriateness of statistical tests used?** Yes, and I have assessed the statistics in my report.

**Comments to author:**

This manuscript elaborates on association of a selected set of SNPs in the mTOR pathway with measures of body composition and additional metabolic phenotypes in humans, followed by in vitro validation of the findings and in vivo experimentation in mouse.

The association of the SNP chosen for functional assay is possible given that it is suggestive in the small size cohort. The concern is that the authors do not provide any validation of this association in the vast data on genetic association studies of common variants in rather large body of literature using significantly larger sample sizes. The authors have successfully undertaken experimental work based on this finding in human studies. The results of these experiments are compelling, and it is possible that the biological mechanisms pointed out by the authors are real. However, it is hard to accept the prediction model that the authors (likely) generated in their discovery cohort without validation in an independent population.

Overall, the manuscript can use a good English language editing to make it compact and effective. Additional specific comments to improve the manuscript are provided below:

Page 3: The graphical abstract is beautiful and captures the essence of the paper well.

Page 4: Line 62: Did the authors intend to use the word "epidemic"? Epidemy is an archaic word that many people will not recognize.

Line 63: The language can be simplified for the definition of obesity. BMI is defined as the ratio between body weight /(height)2, not just a simple ratio as listed.

Regression models: Were any of the study subjects related? How was relatedness tested and what kind of correction was used?

Line 137 - what criterion was used to define the "more restrictive" p-value of 0.01? For a total of 48 SNPs (56 initial with 8 removed for HWE), the Bonferroni correction is 0.001. If this is not being used, the nominal p-value is fine. There is no role of an additional restrictive p-value, unless there is a good reason to justify it.

Lines 143-146 - do the authors mean FDR, rather than Bonferroni? There is only Bonferroni p-value that will be .05/48 = .001 for the SNP association analyses conducted in this manuscript.

Page 7: Significant redundancy in the language in the results section - it can be consolidated for better flow.

Lines 147-152 - when multiple SNPs from one gene are associated with phenotype, one can undertake conditional analyses to identify the SNP with the strongest association. The authors should review their data to assess if this can be done.

Line 163 - This is confusing. The authors state that they removed 8 SNPs for their analyses. If this is true, they should report the actual number of SNPs that were eventually used in analyses, not the overall SNPs. There is little difference between 56 and 48 (both far away from genome wide). So, it is better to report what they used.

Line 195 - Please expand the acronym COSMIC? what proportion of the 965 COSMIC cell lines were minor homozygous (TT)?

Line 201 - Were all the 161 healthy volunteers have homozygous TT? If not, would the authors please provide the proportion of the different genotypes? This reviewer questions the utility of the FNIP2 PBMC expression experiment based on the barely significant p-value that disappears when adjusted for covariates.

Lines 204-208 - please provide the p-values in addition to the r2. What are the '89 healthy volunteers' in this experiment? Are these from the 161 used in the FNIP2 expression experiment? Why are the numbers discordant?

Table S3: This appears to be the association of gene expression levels with the phenotype. Was it for 161 or 89 subjects? Can the authors please provide phenotype data for this cohort? They can probably combine this phenotype data in a new column for Table S1.

Line 250 - what is RPMI? Please expand the acronym. Similarly, TFEB band in line 256.

Line 306 - multifactorial genetic model - which cohort was used to construct this model? If they used the same cohort as their discovery cohort, it is likely extreme overfitting.

A model of this nature needs to be either built in an independent population or to the very least validated in an independent cohort. The validity of such a predictive model is highly questionable.

Line 349 - The "corrected" p-value (how, not known) for rs2291007 with fat mass is .03, and with muscle mass is .02. It is a stretch to call this "strong" association.

Need titles for all supplementary tables.

Supplementary table 1: The authors need to provide reference data for most of the variables.

Check the distribution of the variable and pick either mean or median - both are not needed. In most of these, the mean and median are the close or similar. If the data are normally distributed, please use mean and 95% CI

Sex distribution, age, height, and weight are descriptive.

Fat mass percentage, muscle mass percentage - what range is considered normal for a reference population comparable to those used in this study? Please include these details in the table.

Visceral fatness classification - what is the scale for this classification? This is not a standard measure by any means.

Waist and hip circumference differs by sex. What do they consider as normal range?

Systolic and diastolic BP - what is the normal range? The mean and median are higher than normal in most populations. Do they really have someone with SBP of 12 and DBP of 7 mm Hg? Is this biologically plausible in an adult? HR range of 37 beats/min? Is this biologically plausible? Pulse has no meaning in this context, really.

All the labs need reference ranges. Was the glucose and insulin fasting?

What is the scale of the "Apetite"? How was it measured, what does it mean for a free population, when there is no comparison with obese or other phenotype?

Same applies to calories, basal metabolism and exercise.

Table S3: what is the p-value adjustment used?

Table S4: What does p.val.MT mean? These abbreviations likely mean something to the authors but do not mean anything to a new reader.

**Authors Response**

**Point-by-point responses to the reviewers’ comments:**

**Reviewer 1:**

This study described in this manuscript addresses FNIP2 polymorphism in obesity-related phenotypes, based on the authors' observation that a single nucleotide polymorphism in FNIP2 is associated with obesity state in human. The data show that rs2291007, a T-to-C variant at the 3' UTR region of FNIP2 in increases FNIP2 mRNA stability. In human and a mouse model this increase in FNIP2 expression correlates with a decreased adiposity and increased leanness. This is an interesting study that supports a potential use of FNIP2 and FLCN levels in peripheral blood as a biomarker for body mass index prediction. It also bears an important implication for the function of the FLCN-FNIP complex controlling adipogenesis and body fat deposition. The manuscript is well-organized, and data clearly presented, nevertheless, there are a couples of issues need clarification.

*Response: We thank the reviewer for the enthusiastic consideration of our work.*

1. While data show that miR-181b-5p binds to the T allele of FNIP2, there is no evidence that the miRNA contributes to obesity-related phenotypes in human and the animal model. The authors should include a discussion about the potential role of the miRNA in metabolic phenotypes.

*The reviewer raises an interesting point for discussion. The differential effect of miR181b-5p on the T vs. C rs2291007 alleles is central in our model. While the molecular model does not require changes in the levels of miR-181b-5p, and in the assayed conditions with samples from human and mice we found no evidence of such changes, this is certainly possible. Indeed, changes in miR-181b-5p have been documented in in mouse models of obesity (ob/ob) and diabetes (db/db) and in diabetic individuals [1]. Following the reviewer’s suggestion, we have now discussed the potential role of the miRNA in metabolic phenotypes, emphasizing that either transient, moderate and/or cell-specific fluctuations in miR-181b-5p may contribute to metabolic phenotypes.*

2. Are there associations of FLCN and FNIP2 levels with aging or sex?

*We have tested potential associations by using simple linear models, and found that only FNIP2 displays significant associations with age. The estimated slopes and pvalues are: Age (beta and 95%CI): FNIP2: -0.0227(-0.0352, -0.0101), p-value 4.69E-04, FLCN: 0.0074(-0.0027,0.0175), p-value 0.15. Sex (beta and 95%CI): FNIP2: 0.352(-0.0209,0.725), p-value 0.064, FLCN: -0.282(- 0.573,0.00993), p-value 0.058. Nevertheless, in our analysis, individual associations of FLCN and FNIP2 levels with phenotypic characteristics were adjusted by age and sex, which means that the observed effect is independent of both variables. In the multifactorial model, sex and age have been computed as covariates, so the effect of the gene expression and SNP on BMI is adjusted for these.*

3. Figure 2C, does lower half of the figure depict miR-181a -5p binding or is a typo?

*We thank the reviewer for pointing out this mistake. We have changed Figure 2C accordingly.*

**Reviewer 2:**

This manuscript elaborates on association of a selected set of SNPs in the mTOR pathway with measures of body composition and additional metabolic phenotypes in humans, followed by in vitro validation of the findings and in vivo experimentation in mouse.

The association of the SNP chosen for functional assay is possible given that it is suggestive in the small size cohort. The concern is that the authors do not provide any validation of this association in the vast data on genetic association studies of common variants in rather large body of literature using significantly larger sample sizes. The authors have successfully undertaken experimental work based on this finding in human studies. The results of these experiments are compelling, and it is possible that the biological mechanisms pointed out by the authors are real.

*Response: We are thankful to the reviewer for the positive remarks on our work, for a thorough assessment of the data, and we appreciate his/her valuable comments and suggestions*.

However, it is hard to accept the prediction model that the authors (likely) generated in their discovery cohort without validation in an independent population.

*We agree with the reviewer on the value of testing our model in an independent population, however we must also point out the difficulty in obtaining a new and similarly large set of samples from healthy volunteers. We attempted to obtain comparable datasets from published work, but failed to identify studies encompassing genetic data, clinical parameters, and fundamentally, gene-expression data from peripheral blood to replicate our conclusions. Aware of the limitation raised by the reviewer, in our original submission we purposedly challenged our statistical analysis with stringent bootstrapping, as follows: we randomly resampled the whole cohort, rederived the model with each resampling event, and re-tested it including those subjects not used in the original fitting. This process was repeated 2000 times, and corrections for error prediction were performed as defined by standard bootstrap prediction errors. Bootstrapping methodologies to correct for overfit in predictive models are well established, both theoretically and in practice [2,3]. In our predictive model, such methodology allowed us to correct the performance by optimism, and to assess its predictive power and possible overfit, so as to estimate future performance in external datasets. In addition to this bootstrap validation, we have included a brief discussion acknowledging the intrinsic limitation of this modelling, and the robustness of bootstrapping methodology. We hope this reviewer understands the intrinsic difficulties of finding comparable cohorts and shares our vision of the future independent validation efforts that will be spurred by our work.*

Overall, the manuscript can use a good English language editing to make it compact and effective.

*We thank the reviewer, and have performed English language editing to improve readability.*

Additional specific comments to improve the manuscript are provided below:

Page 3: The graphical abstract is beautiful and captures the essence of the paper well.

*We are happy that our effort in providing both beauty and scientific content is appreciated.*

Page 4: Line 62: Did the authors intend to use the word "epidemic"? Epidemy is an archaic word that many people will not recognize.

*We have removed the word “epidemy” from the main text.*

Line 63: The language can be simplified for the definition of obesity. BMI is defined as the ratio between body weight /(height)2, not just a simple ratio as listed.

*We amended the mistake and condensed the sentence.*

Regression models: Were any of the study subjects related? How was relatedness tested and what kind of correction was used?

*The reviewer raises an important point for clarification. The subjects included in the regression models were kinship-unrelated (i.e.: no siblings, no siblings-parents’ pairs, etc.) and randomly sampled from the Spanish population. Therefore, genetic distribution of samples is expected to be randomly obtained from the population and with no significant relatedness, particularly taking into account the size of the sample. The small number of non-equilibrium Hardy-Weinberg SNPs, together with the use of SNPs only in Hardy-Weinberg equilibrium, provides strong support for the absence of population admixture and/or stratification regarding the SNPs.*

Line 137 - what criterion was used to define the "more restrictive" p-value of 0.01? For a total of 48 SNPs (56 initial with 8 removed for HWE), the Bonferroni correction is 0.001. If this is not being used, the nominal p-value is fine. There is no role of an additional restrictive p-value, unless there is a good reason to justify it.

*We must admit that “more restrictive” is an unacceptable statistical criterion. We thank the reviewer for pointing this out, and agree with him/her that nominal p-value is correct. We have amended the text and Figure accordingly.*

Lines 143-146 - do the authors mean FDR, rather than Bonferroni? There is only Bonferroni p-value that will be .05/48 = .001 for the SNP association analyses conducted in this manuscript.

*We apologize for the lack of clarity with “Bonferroni p-value” when referring to the pvalue of the test after applying Bonferroni correction to the original p-value, namely: min (1, pval*n.tests). A threshold p-value for significance (equivalent to the typical 0.05 threshold for a single test) in this 48-test situation is 0.05/48, but a Bonferroni-corrected p-value (min (1, pval*48) informs on significance of the test after applying a multipletest correction, using the accepted 0.05 threshold. Nevertheless, taking into consideration the reviewer’s comment, we have changed “Bonferroni p-value” for “Bonferroni-corrected p-value”.*

Page 7: Significant redundancy in the language in the results section - it can be consolidated for better flow.

*We believe to have improved the flow and increased the precision of the wording*

Lines 147-152 - when multiple SNPs from one gene are associated with phenotype, one can undertake conditional analyses to identify the SNP with the strongest association. The authors should review their data to assess if this can be done.

*We agree with the reviewer. Conditional analyses are appropriate to identify the strongest association. Conditioning has been tested by means of developing predictive models for fat mass as response, and adjusted for sex and age. Importantly, a model with rs10857319 as predictor variable did not reach statistical significance when including the SNP rs2291007. An identical result was obtained for a model with rs17286116 when including the SNP rs2291007. We have modified the text as follows:*

*“We further analyzed the three SNPs detected in FNIP2 genomic region (rs10857319, rs17286116 and rs2291007), located in 5´UTR, intronic and 3´UTR regions, respectively (Fig 1B and Supplementary Table 2). rs17286116 and rs2291007 share linkage disequilibrium, with a R2 value of 0.735 (Fig 1B), explaining the overlapping associations detected. rs2291007, located in FNIP2 3´UTR region, had the highest beta and lowest p-values. To identify the strongest associations, we have performed conditional analyses by developing predictive models for fat mass, adjusted for sex and age. Those analyses identified rs2291007 as the strongest independent signal for this locus, because adding the rs2291007 SNP to the analysis resulted in the other two SNPs (rs10857319 and rs17286116) losing statistical significance.”*

Line 163 - This is confusing. The authors state that they removed 8 SNPs for their analyses. If this is true, they should report the actual number of SNPs that were eventually used in analyses, not the overall SNPs. There is little difference between 56 and 48 (both far away from genome wide). So, it is better to report what they used.

*The reviewer is correct, and analyses were conducted with 48 SNPs. Thus, we have modified the text to reflect that 48 is the number of loci ultimately used in the analyses, not 56. We thank the reviewer for pointing out this confounding mistake.*

Line 195 - Please expand the acronym COSMIC? what proportion of the 965 COSMIC cell lines were minor homozygous (TT)?

*We have explained the COSMIC acronym for Catalogue of Somatic Mutations in Cancer in the text. We have also added information on the proportion of cell lines with the T/T genotype. From a total of 965 cell lines, 446 (46.2%) were C/C, 305 (31.6%) were C/T and 214 (22.2%) were T/T for rs2291007.*

Line 201 - Were all the 161 healthy volunteers have homozygous TT? If not, would the authors please provide the proportion of the different genotypes? This reviewer questions the utility of the FNIP2 PBMC expression experiment based on the barely significant p-value that disappears when adjusted for covariates.

*Analysis of FNIP2, FLCN and FNIP1 expression in PBMCs were performed in 161 healthy volunteers of the three genotypes: 22.98% CC, 51.55% C/T and 25.46% T/T. We have added genotype proportions to the main text. The expression data (in an arguably reduced number of volunteers’ samples) does not reach statistically significant p-value, but the p=0.1 trend goes in line with the idiosyncrasy of the paper, and more so with the strong, significant association of FNIP2 expression with metabolic characteristics related to obesity phenotypes.*

Lines 204-208 - please provide the p-values in addition to the r2. What are the '89 healthy volunteers' in this experiment? Are these from the 161 used in the FNIP2 expression experiment? Why are the numbers discordant?

*We have added p-values of simple lineal regression in the text and in Supplementary Figure 3A. We regret the lack of clarity with the source of these biological samples in our original submission. Our collection includes purified mRNA from 161 healthy volunteers, but because isolation of microRNA requires specific extraction methods, microRNAs were only available from 89/161 volunteers. We have added this information to the text.*

Table S3: This appears to be the association of gene expression levels with the phenotype. Was it for 161 or 89 subjects? Can the authors please provide phenotype data for this cohort? They can probably combine this phenotype data in a new column for Table S1.

*We believe the reviewer is referring to Supplementary Table 4 here. Supplementary Table 4 summarizes genetic information on rs number, genes, beta, 95% CI and pvalues for each metabolic association in the entire cohort of 790 healthy volunteers. Supplementary Table 4 shows the association of gene-expression levels with the phenotype. Following this reviewer’s suggestion, we have now included two additional columns on Supplementary Table 1: -Phenotype data for the 161 individuals. -Phenotype data for the subset of 89 individuals used for the analysis of microRNA expression This addition is accompanied by an explanation in the main text.*

Line 250 - what is RPMI? Please expand the acronym. Similarly, TFEB band in line 256.

*We have amended the text.*

Line 306 - multifactorial genetic model - which cohort was used to construct this model? If they used the same cohort as their discovery cohort, it is likely extreme overfitting.

A model of this nature needs to be either built in an independent population or to the very least validated in an independent cohort. The validity of such a predictive model is highly questionable.

*We developed a multifactorial statistical model including gene-expression variables plus sex and age, in addition to the rs2291007 SNP, following standard methodology. We agree with the reviewer that the use of same cohort could easily result in overfitting, and our awareness of this undesirable possibility prompted us to conduct bootstrap validation to correct for potential overfitting. As stated on page 2 of this rebuttal letter, we randomly resampled the original cohort, derived the model with the resampled subjects, and used this model to make inference for entire cohort. This exercise was repeated 2000 times. Thus, this bootstrapping provides some degree of correction to potential overfitting, and in spite of this, the corrected model displays reasonable predictive power. Importantly the use of bootstrap methodologies to correct for overfit in predictive models is well established in statistical modeling, both theoretically and in practice [2,3] and are of paramount importance when validation cohorts cannot be obtained. We need to emphasize the difficulty in obtaining a new and independent set of samples: this population is a healthy and non-pathologic population that participates voluntarily, and obtained a 6-year period (2012-2018). Thus, the present collection of DNA, RNA and microRNA specimens, together with phenotypic and anthropometric data, is extremely hard to obtain and replicate in a short period of time. Based on the results present in the manuscript, we expect that ourselves, and also others, will aim to perform additional clinical studies in healthy and in pathologic populations. As mentioned above, we have now discussed a discussion on limitations and future promise of the multifactorial model.*

Line 349 - The "corrected" p-value (how, not known) for rs2291007 with fat mass is .03, and with muscle mass is .02. It is a stretch to call this "strong" association.

*We have toned down the statement and deleted the term “strong”, and as commented above (comment on Line 143-146), also clarified the “corrected” p-value.*

Need titles for all supplementary tables.

*We thank the reviewer for pointing out this omission; we have included titles in Supplementary Tables.*

Supplementary table 1: The authors need to provide reference data for most of the variables.

Check the distribution of the variable and pick either mean or median - both are not needed. In most of these, the mean and median are the close or similar. If the data are normally distributed, please use mean and 95% CI

*We now provide reference data for phenotypic variables in Supplementary Table 1. Because all variables are normally distributed, we now show 95% CI and mean as indicated*

Sex distribution, age, height, and weight are descriptive. Fat mass percentage, muscle mass percentage - what range is considered normal for a reference population comparable to those used in this study? Please include these details in the table.

*As described in Methods section, weight (kg), fat mass (%), and muscle mass (%) were estimated using bioelectrical impedance analyses (Body Composition Monitor BF511-OMRON HEALTHCARE, LT, Kyoto, Japan). Percentage fat mass and percentage muscle mass ranges considered normal for a reference population vary as a function of age and sex. Importantly, associations for rs2291007 were adjusted by sex and age. Below are reference values provided by OMRON manufacturer [4,5].*

Visceral fatness classification - what is the scale for this classification? This is not a standard measure by any means.

*Visceral fatness has been classified by bioimpedance and as per manufacturer's references (BF511 Body Composition Monitor; Omron Healthcare Co., Ltd) [4,5].*

Waist and hip circumference differs by sex. What do they consider as normal range?

*The normal values for waist circumference for both women and men in the Spanish population according to Consenso SEEDO’2000 [6] are <95cm for men and <82cm for women. To our knowledge, there is no standardized reference value for hip circumference.*

Systolic and diastolic BP - what is the normal range? The mean and median are higher than normal in most populations. Do they really have someone with SBP of 12 and DBP of 7 mm Hg? Is this biologically plausible in an adult?

*Normal ranges of arterial pressure are 120-129 for SBP and 80-84 for DBP [7]. In our GENYAL Platform on Nutrition and Health of IMDEA Food Institute, we have reported blood pressure measurements in adults of 120 SBP and 70 DBP mmHg and even lower. Hypotension is frequent in the general population and is associated with a benign cardiovascular risk factor profile [8]. We acknowledge that 12 and 7 mmHg were typos, and the correct numbers, 120 and 70, respectively, are now in Supplementary Table 1. We thank the reviewer for pointing them out.*

HR range of 37 beats/min? Is this biologically plausible? Pulse has no meaning in this context, really.

*Although a heart rate of 60 to 100 beats per minute at rest is considered normal, and our cohort fits with this range, there are healthy subjects with a value that below the normal range.*

All the labs need reference ranges. Was the glucose and insulin fasting?

*We have clarified in the text that blood tests were performed in following overnight fasting. A fasting blood sugar level of 99 mg/dL or lower was considered normoglycemia. Free insulin levels below 17 mcU/mL was considered normal fasting insulinemia.*

What is the scale of the "Apetite"? How was it measured, what does it mean for a free population, when there is no comparison with obese or other phenotype?

*We have now corrected the typo, and more importantly, clarified in the text how was Appetite measured and graded. Self-perceived appetite was graded by means of a questionnaire-based Likert scale with 5 points: 1–2 for low appetite, 3 for intermediate level of appetite, and 4–5 for high appetite.*

Same applies to calories, basal metabolism and exercise.

*These nutritional assessments (diet and physical activity) were collected as previously described [9–12]. There are no reference values in the healthy population in general. Calories consumption was determined in a 72-hour record of food consumption that was collected from all participants. DIAL Software (2.16 version, Alce Ingeniería) [13] was used to infer energy intake (calories), macro- and micro-nutrients from the participants’ records.*

*Basal metabolism was determined using the body composition monitor BF511 (Omron Healthcare UK, 104 LT, Kyoto, Japan).*

*The “exercise” variable was generated as a discrete numeric variable accounting for the weekly frequency of physical exercise. It was a self-assessment of the number of exercise-days per week: 0, 1, 2, 3, 4-5 and > 5 times (Codified from 1 to 6).*

*Collectively, all the parameters and variables explained above, related to Supplementary Figure 1, are now explained in Supplementary Table 1 and Methods Section and also referenced [9–12].*

Table S3: what is the p-value adjustment used?

*Bonferroni multiple-test correction was used: min (1, p.val*number of tests). To clearly state the statistical correction, we have modified and homogenized the headers on Supplementary Tables and Methods Section (Bonferroni corrected p-value).*

Table S4: What does p.val.MT mean? These abbreviations likely mean something to the authors but do not mean anything to a new reader.

*We apologize once again for the lack of clarity. p.val.MT means p-value after Bonferroni multiple-test correction, min (1, p.val*number of tests). We have amended Supplementary Table 4 to clarify this.*

REFERENCES

1. Wang S, Liang C, Ai H, Yang M, Yi J, Liu L, et al. Hepatic miR-181b-5p Contributes to Glycogen Synthesis Through Targeting EGR1. Dig Dis Sci. 2019;64:1548–59.

2. Efron B, Hastie T. Computer Age Statistical Inference: Algorithms, Evidence, and Data Science [Internet]. 1st ed. Cambridge University Press; 2016 [cited 2021 Nov 12]. Available from: <https://www.cambridge.org/core/product/identifier/9781316576533/type/book>

3. Harrell , FE. Regression Modeling Strategies: With Applications to Linear Models, Logistic and Ordinal Regression, and Survival Analysis [Internet]. Cham: Springer International Publishing; 2015 [cited 2022 Jul 26]. Available from: <http://link.springer.com/10.1007/978-3-319-19425-7>

4. McCarthy HD, Cole TJ, Fry T, Jebb SA, Prentice AM. Body fat reference curves for children. Int J Obes (Lond). 2006;30:598–602.

5. Gallagher D, Heymsfield SB, Heo M, Jebb SA, Murgatroyd PR, Sakamoto Y. Healthy percentage body fat ranges: an approach for developing guidelines based on body mass index. Am J Clin Nutr. 2000;72:694–701.

6. Consenso SEEDO’2000 para la evaluación del sobrepeso y la obesidad y el establecimiento de criterios de intervención terapéutica. 2000;115:11.

7. Mancia G, Fagard R, Narkiewicz K, Redón J, Zanchetti A, Böhm M, et al. 2013 ESH/ESC Guidelines for the management of arterial hypertension: the Task Force for the management of arterial hypertension of the European Society of Hypertension (ESH) and of the European Society of Cardiology (ESC). J Hypertens. 2013;31:1281–357.

8. Owens PE, Lyons SP, O’Brien ET. Arterial hypotension: prevalence of low blood pressure in the general population using ambulatory blood pressure monitoring. J Hum Hypertens. 2000;14:243–7.

9. Camblor Murube M, Borregon-Rivilla E, Colmenarejo G, Aguilar-Aguilar E, Martínez JA, Ramírez De Molina A, et al. Polymorphism of CLOCK Gene rs3749474 as a Modulator of the Circadian Evening Carbohydrate Intake Impact on Nutritional Status in an Adult Sample. Nutrients. 2020;12:1142.

10. Marcos-Pasero H, Colmenarejo G, Aguilar-Aguilar E, Ramírez de Molina A, Reglero G, Loria-Kohen V. Ranking of a wide multidomain set of predictors of children obesity by Machine Learning variable importance techniques. Submitted for publication. 2020;

11. Espinosa-Salinas I, San-Cristobal R, Colmenarejo G, Loria-Kohen V, Molina S, Reglero G, et al. Polymorphic Appetite Effects on Waist Circumference Depend on rs3749474 CLOCK Gene Variant. Nutrients. 2020;12:1846.

12. Espinosa-Salinas I, de la Iglesia R, Colmenarejo G, Molina S, Reglero G, Martinez JA, et al. GCKR rs780094 Polymorphism as A Genetic Variant Involved in Physical Exercise. Genes. 2019;10:570.

13. Ingenieria A. DIAL Programa para Evaluación de Dietas y cálculos de Alimentación. :132.

**Second round of review**

**Reviewer 1**

*The authors have adequately addressed my comments as well as those of the other reviewer. The manuscript is now suitable for publication.*
